# Direct Observation of the Dynamics of Ylide Solvation by Hydrogen-bond Donors Using Time-Resolved Infrared Spectroscopy

**DOI:** 10.1021/jacs.2c01208

**Published:** 2022-05-17

**Authors:** Ryan Phelps, Andrew J. Orr-Ewing

**Affiliations:** School of Chemistry, University of Bristol, Cantock’s Close, Bristol BS8 1TS, U.K.

## Abstract

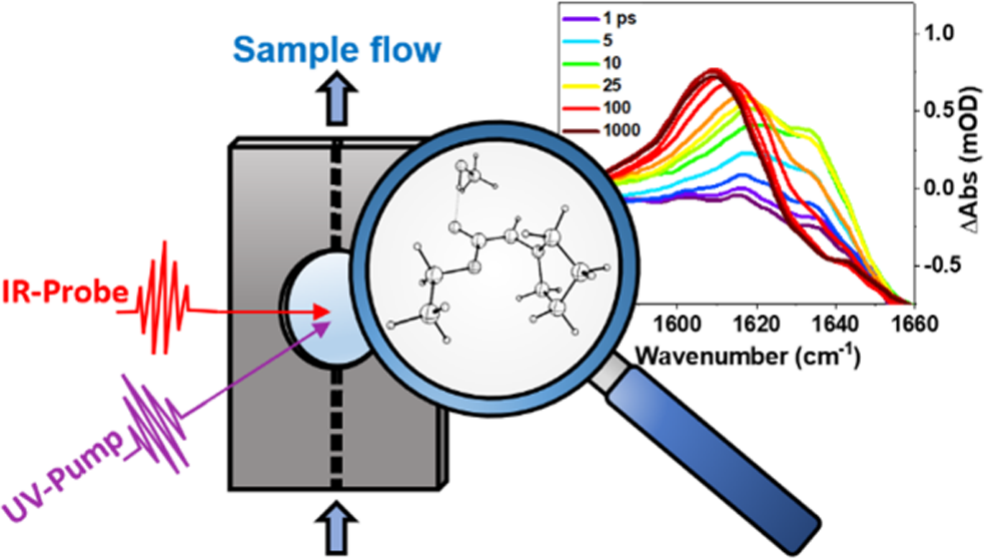

The photoexcitation of α-diazocarbonyl compounds produces singlet carbene intermediates that react with nucleophilic solvent molecules to form ylides. The zwitterionic nature of these newly formed ylides induces rapid changes in their interactions with the surrounding solvent. Here, ultrafast time-resolved infrared absorption spectroscopy is used to study the ylide-forming reactions of singlet carbene intermediates from the 270 nm photoexcitation of ethyl diazoacetate in various solvents and the changes in the subsequent ylide–solvent interactions. The results provide direct spectroscopic observation of the competition between ylide formation and C–H insertion in reactions of the singlet carbene with nucleophilic solvent molecules. We further report the specific solvation dynamics of the tetrahydrofuran (THF)-derived ylide (with a characteristic IR absorption band at 1636 cm^–1^) by various hydrogen-bond donors and the coordination by lithium cations. Hydrogen-bonded ylide bands shift to a lower wavenumber by −19 cm^–1^ for interactions with ethanol, −14 cm^–1^ for chloroform, −10 cm^–1^ for dichloromethane, −9 cm^–1^ for acetonitrile or cyclohexane, and −16 cm^–1^ for Li^+^ coordination, allowing the time evolution of the ylide–solvent interactions to be tracked. The hydrogen-bonded ylide bands grow with rate coefficients that are close to the diffusional limit. We further characterize the specific interactions of ethanol with the THF-derived ylide using quantum chemical (MP2) calculations and DFT-based atom-centered density matrix propagation trajectories, which show preferential coordination to the α-carbonyl group. This coordination alters the hybridization character of the ylidic carbon atom, with the greatest change toward sp^2^ character found for lithium-ion coordination.

## 1 Introduction

The synthesis of numerous fine chemicals, pharmaceutical compounds, and agrochemicals relies on reactions in organic solvents, but the choice of solvent can affect the thermodynamics and kinetics, and hence the outcomes of chemical reactions. In the solutions used for synthesis, solute–solvent interactions will influence the rates, product yields, and selectivity of the chemistry.^1−3^ Many solvent-induced effects can be attributed to bulk properties such as the solvent’s dielectric constant, for example, polar solvents stabilize polar or charged intermediates and nucleophiles. However, these bulk properties are not always sufficient to account for solvent-induced trends in chemical reactivity because at the molecular level, specific solute–solvent interactions can be significant. Such behavior is common for protic solvents, including water and alcohols, for which the influence of hydrogen bonding must be considered to understand how the solvent influences a reaction.^4−6^ Efforts to unravel the effects of hydrogen bonding on the stability, molecular structure, and reactivity of reaction intermediates in solution are therefore important across chemistry, as well as in biochemistry.

Zwitterions are dipolar neutral molecules carrying both formal negative and positive charges, and this polarity makes them particularly sensitive to their solvent environment.^7−10^ Zwitterions come in many forms, for example, ylides and betaines are frequently invoked, short-lived intermediates for a variety of biochemical transformations^11−13^ and synthetic reactions such as the Wittig olefination.^14−18^ Many synthetically useful ylide transformations use metal catalysts,^19^ but efforts are now being made to work under metal-free conditions.^20,21^ Because of the utility of such reactions, ylide reactivities have been extensively studied.^20−24^ Examples include several mechanistic investigations of the Wittig olefination by the reaction of phosphonium ylides with carbonyl compounds.^17,25−27^ In such reactions, the product stereochemistry depends on the stability of the ylide,^17,25,28−36^ with stabilized ylides showing greater E selectivity in the alkene products. Although protic solvents are known to alter reaction rates and selectivity in a series of Wittig reactions, the solvent-induced effects depend on the ylides involved.^30−36^ Ayub and Ludwig provided some insights into these interactions by computing complexes of a gas hydrate consisting of 20 water molecules with an α-carbonyl phosphorus ylide and the transition states for the cis and trans reaction pathways of the ylide with benzaldehyde.^37^ They found that hydrogen bonds (HBs) donated from the gas hydrate stabilized the transition states relative to the reagents for both addition pathways but with greater stabilization for the cis-reaction. These results suggest ylide reaction rates will be faster in aqueous media compared to the more weakly interacting organic solvents but with reduced selectivity toward the E-isomer product formed from the trans-addition pathway. Experimentally, reaction times are reduced in water compared to organic solvents but with no substantial change to the stereochemistry of the resulting products.^32^

The effects of solvation on other ylide-mediated reactions have also been investigated. For example, Lee et al. studied the effect of the solvent on sulfur-ylide-mediated epoxidation and showed that the addition of 0.15 M methanol in nonpolar solvents changed the stereochemical outcomes, which they argued to be a result of specific betaine–methanol interactions.^38^ Dontsova et al. explored the effect of solvent on the regioselectivity of the reactions of pyridinium ylides with alkenes and found that protic solvents changed the reaction pathway.^39^ Using both experimental and computational methods, Biswas and Singleton examined how hydrogen bonding affects the competition between [1,2] and [2,3]-sigmatropic rearrangements in α-carbonyl ylide-mediated reactions. HB donation to the α-carbonyl group in an ammonium ylide was shown to promote the [2,3]-rearrangement pathway because of its influence on the location of the reaction transition state.^40^ Further examples of reaction control using α-carbonyl ylides in protic solvents have also been reported.^41,42^ These and other prior investigations demonstrate a need to probe directly the interactions of different solvents with various zwitterionic intermediates.

Several theoretical and computational studies have examined ylides as hydrogen-bond acceptors.^43−46^ For example, Rozas et al. and Platts and Howard showed that ylide complexes could be formed with a series of strong and weak HB donors. Their calculations identified that even the weak HB donor C_2_H_2_ could form stable ylide complexes with HB strengths of 16–34 kJ mol^–1^,^44,45^ and CH_4_ formed such HBs with binding energies of 3–5 kJ mol^–1^.^45^ Further evidence for these types of C–H HBs with ylides came from the crystal structure of triphenylphosphonium benzylide, which showed a pairwise alignment suggestive of C–H HB donation to an acceptor atom in the ylide.^43^ Despite the propensity for ylides to act as HB acceptors, direct experimental evidence of solvent–ylide complexes is scarce, as is evidence for the structural and electronic changes that result from hydrogen-bonded complexation.

The presence of cations in solution can also influence reaction pathways passing through zwitterionic species. For example, lithium salts are commonly used additives for Wittig reactions involving phosphonium ylides and have been found to alter reaction rates and the selectivity of products.^17,47,48^ The lithium salt effect in Wittig reactions has been attributed to the stabilization of betaine intermediates formed by the reaction of phosphonium ylides with carbonyl compounds.^17,48^ In their studies of the lithium salt concentration dependence of reaction rates and product yields for Wittig reactions in tetrahydrofuran (THF), Reitz et al. found a hyperbolic relationship to the E/Z-isomer product ratios.^47^ They argued that THF molecules compete in solvating the Li^+^ ions and therefore, only at higher concentrations of added LiBr are Li^+^ ions free to interact with the betaine intermediates. Prior observations of lithium-ion-stabilized zwitterionic intermediates used low temperatures to isolate a betaine–LiBr adduct.^49^ Although the mechanism of the Wittig reaction is now well characterized, a full understanding of the involvement of Li^+^ cations remains to be established.

**Scheme 1:**
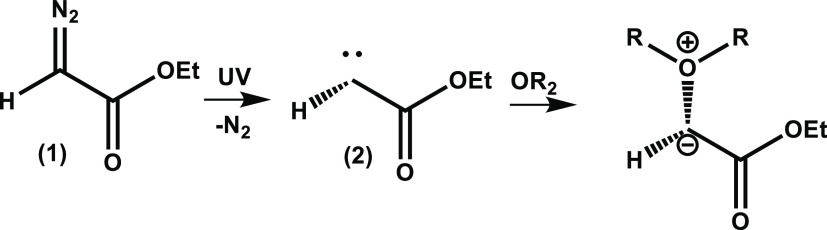
Photolytic Decomposition of Ethyl Diazoacetate (**1**) to Produce Singlet α-Carbonyl Carbene Intermediate (**2**) and an Example Reaction Pathway of Carbene (2) with an Ether to Form an α-Carbonyl Ylide

**Scheme 2:**
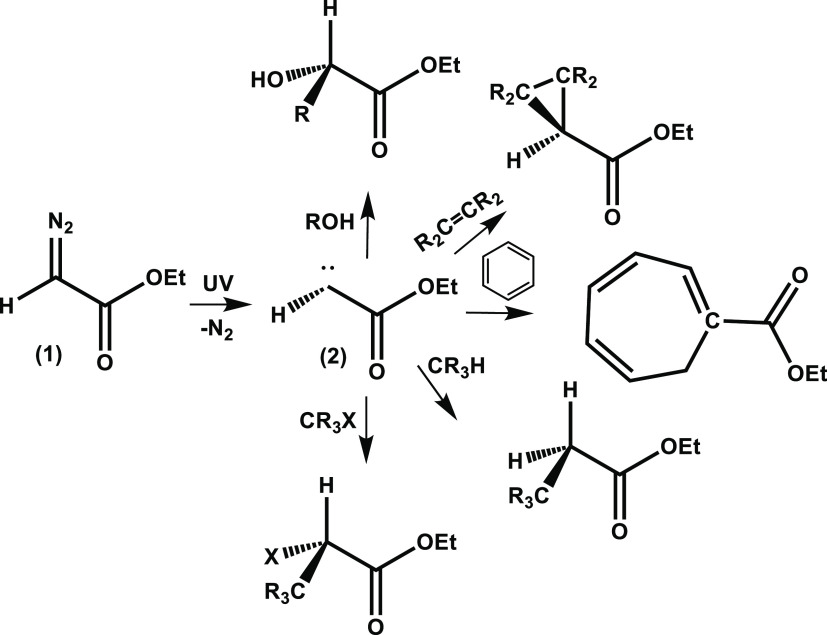
Photolytic Decomposition of Ethyl Diazoacetate (**1**) to Produce a Singlet α-Carbonyl Carbene Intermediate (**2**) and Example Insertion and Addition Reaction Pathways of the Singlet Carbene

Here, we study the specific interactions of α-carbonyl ylides with different solvent molecules and with Li^+^ ions to explore the consequences for the ylide structures and stabilities. A convenient way to produce these ylides in solution is by the reaction of carbenes with nucleophilic solvents such as alcohols, ethers, or acetonitrile (ACN), as is illustrated in Scheme 1 for the reaction of a carbene (2) with an ether. α-Diazocarbonyl compounds, such as ethyl diazoacetate (EDA, (1)) can be used as precursors for singlet α-carbonyl carbenes, which form by photolytic elimination of N_2_ (Scheme 1).^50^ In competition with ylide formation, these carbenes can add to, or insert into, a variety of functional groups, such as C=C, C–H, C–X (X = Cl, Br, I), aromatic rings, and O–H bonds, as shown in Scheme 2,^51,52^ with the mechanism of insertion typically inferred from the stereochemistry of the products. The direct observation of carbene reaction pathways has been the subject of several previous studies.^53−61^ By monitoring the C=O stretching modes using time-resolved infrared (TRIR) spectroscopy,^54^ Xue et al. demonstrated that a variety of ylides could be directly observed from the reaction of the singlet α-carbonyl carbene (2) with nucleophilic solvents such as THF, ACN, and deuterated methanol (MeOD). Similarly, we recently assigned bands observed by TRIR to ylides produced following the photoexcitation of ethyl diazoacetoacetate in several aprotic nucleophilic solvents. In addition, we identified the involvement of the α-carbonyl group in the reactivity of the α-dicarbonyl carbene with alcohols and provided experimental and computational evidence for an enol-forming pathway that bypasses the alcohol ylide.^55^ Ylide-forming reaction pathways have also been reported for carbenes that do not bear an α-carbonyl group.^56,57^

Solvation of carbenes has been the focus of several prior experimental and theoretical studies,^62−69^ but the solvation dynamics of the ylide intermediates of carbene reactions have yet to be examined. Using band shifts observed by TRIR, we present direct spectroscopic evidence for specific solvent interactions between EDA-derived THF ylides (THF-Y) and ethanol (EtOH), chloroform, dichloromethane (DCM), ACN, and cyclohexane HB donors, and we characterize the kinetics of solvation. In addition, we use TRIR to explore the influence of lithium-ion coordination. In combination with quantum chemical calculations and dynamical trajectory simulations, our TRIR measurements identify the structural changes to the α-carbonyl ylide intermediates induced by the solvent complexation, and they provide new insights into how ylide–solvent and ylide-Li^+^ interactions affect the ylide-mediated reaction mechanisms.

## 2 Methods

### 2.1 Experiment

The photoinduced chemistry of ethyl diazoacetate (1) was studied in several solvents and solvent mixtures using ultrafast TRIR with a broadband IR probe. Spectra were collected following the photoexcitation at 270 nm of 65 mM samples of EDA (Sigma-Aldrich, ≤100%) used as received. Absorption at 270 nm is assigned to the S_2_ ← S_0_ excitation with ππ* character to the diazo functional group. With a sample path length of 150 μm, an absorbance (optical density, OD) at 270 nm <0.8 was obtained. Measurements were made for EDA solutions in cyclohexane (Fisher Scientific, extra pure, SLR grade), tetrahydrofuran (Fisher Scientific, extra pure, SLR grade, stabilized with 0.025% BHT), acetonitrile (Fisher Scientific, HPLC gradient grade, <99.9%), ethanol (Sigma-Aldrich, ACS reagent grade, >99.5%), methanol (ACS reagent grade, >99.5%), dichloromethane (Analytical grade, ≥99%), and chloroform (anhydrous, ≥99%, stabilized with <1% ethanol). Steady-state FTIR and UV absorption spectra for these EDA solutions are shown in Figures S4 and S5 of the Supporting Information (SI).

The TRIR instrumental setup has been described in detail elsewhere,^70^ and only key experimental details are reported here. Flowing samples passed through a Harrick cell with CaF_2_ windows separated by 150 μm and were intersected by pulses of 270 nm UV radiation of duration ∼100 fs. After a delay of up to 1.3 ns, controlled by an optical delay line, the UV-excited samples were probed by spatially overlapping mid-IR pulses of duration ∼100 fs and 300 cm^–1^ bandwidth, which were then dispersed in a spectrometer (HORIBA Scientific, iHR320) fitted with a 128-element mercury–cadmium–telluride array detector (Infrared Associates Inc., MCT-10–128) and fast read-out electronics (Infrared Systems Development Corp, FPAS-0144). The mid-IR spectral resolution was 2 cm^–1^ per pixel for spectra acquired around 1650 cm^–1^. A portion of the IR beam was split from the probe beam pathway before the sample and passed to a matched spectrometer to obtain a reference spectrum of each probe pulse. This reference was used in the processing of the TRIR data to reduce the shot-to-shot noise in the transient spectra.

A mechanical chopper (Thorlabs, MC2000) intersected the pump pulse at a repetition rate of 500 Hz to collect pump-on and pump-off spectra with alternate probe laser pulses, from which pump-induced difference spectra were derived. Spectra were obtained under aerobic conditions because the measurement timescales were too short for dissolved oxygen to affect the observed photochemistry.^71^

### 2.2 Computational Details

All calculations were performed using the Gaussian 09 computational package.^72^ Assignments of transient bands in TRIR spectra were supported by scaled harmonic vibrational frequencies computed at the MP2 level using a 6-311++G(d,p) basis set for the majority of the species considered in this study. A frequency scaling factor of 0.9827 was determined from anharmonically corrected vibrational frequency calculations of EDA, which reproduced the carbonyl stretching frequency to within 9 cm^–1^ in cyclohexane, and this factor was applied to all other species. This anharmonic correction factor is in agreement with recent benchmarking data for the carbonyl stretching modes in a series of organic compounds.^73^

To explore the complexation of alcohols to ylides and to guide the interpretation of experimental measurements, structures and vibrational frequencies of hydrogen-bonded THF-ylide-alcohol complexes were calculated. These calculations used methanol as the HB donor in place of the ethanol used in our experiments to reduce the computational demands. Binding energies were computed for various methanol complexes with the THF-ylide using MP2/6-311++G(d,p) geometries and MP2/Aug-cc-pVTZ single point energies. The large basis set was chosen to minimize the consequences of basis set superposition error. The THF bath was included using a polarizable continuum model (PCM). Atom-centered density matrix propagation (ADMP) trajectories were calculated for the various complexes using the B3LYP functional and Grimmes’s D3 empirical correction for dispersion^74^ (B3LYP-D3) with the 6-31+G(d) basis set, as well as inclusion of the THF bath using a PCM. A step size of 0.25 fs was used until the hydrogen bond length exceeded 3.1 Å or for a maximum propagation time of 5 ps for calculations with the B3LYP functional or 4 ps with the B3LYP-D3 functional. The fictitious electron mass was set to 0.1 amu. All trajectories were simulated at 300 K, with a constant temperature maintained by a Gaussian isokinetic thermostat and were computed for the hydrogen-bonded complex structures optimized at B3LYP/6-31+G(d) and B3LYP-D3/6-31+G(d) levels of theory. To extract typical behavior, 25 trajectories were computed for complexes 1 and 2, and 40 trajectories for complex 3 (described later) with randomized starting conditions. We recognize that more trajectories are required to assess typical behavior statistically but were limited by the computational cost of running such calculations.

## 3 Results and Discussion

We begin by outlining our findings for the photoexcitation of EDA dissolved in various solvents before turning to ylide solvation dynamics in binary solvent mixtures. Sections 3.1–3.2 and Section S1 of the SI discuss the different reaction pathways observed in the chosen solutions, which may be common to more than one solvent. Sections 3.3–3.7 then describe the observed solvation and Li^+^ ion complexation dynamics and the associated changes to the electronic structure of the ylide. To facilitate the discussion of the dynamical processes revealed by our TRIR measurements, Table 1 provides a summary of exponential time constants associated with the growth (and, in some cases, subsequent decay) of the initial products of the photochemical pathways identified. Experimental TRIR data for the C–H insertion and enol pathways included in Table 1 are presented in Sections S1.1–S1.2 of the SI. Section S3 of the SI describes the methods used to account for the effects of ground-state bleach recovery and vibrational cooling, which also occur on few-picosecond timescales, in our analysis of TRIR band intensities to extract the reported time constants.

**Table 1:** Band Positions and Time Constants for Intermediate and Product Species Identified Following the 270 nm Photoexcitation of EDA Solutions in Five Solvents

species	band wavenumber (cm^–1^)	τ_1_ (ps)	τ_2_ (ps)
Cyclohexane			
C–H insertion product	1745	12.6 ± 0.4	
THF			
C–H insertion products	1739	6.5 ± 0.3	
(3) THF-Y^a^	1636	6.5 ± 0.3	
ACN			
(4) ACN-Y^a^	1642	8.4 ± 0.3	
C–H insertion product	1740	8.4 ± 0.3	
EtOH			
(5) EtOH-Y^a^	1617	6.1 ± 0.4	121 ± 10
(6) E-Enol	1722	6.1 ± 0.4	
(7) Z-Enol	1722		121 ± 10
(8) Ether	1745		121 ± 10
(9) C–H insertion products	1735	6.1 ± 0.4	
MeOH			
MeOH-Y^a^	1618	5.1 ± 0.4	26 ± 3
E-Enol	1718	5.1 ± 0.4	
Z-Enol	1718		26 ± 3
Ether	1745		26 ± 3

a Solvent-Y denotes a solvent–ylide.

### 3.1 Ylide Formation

**Figure 1:**
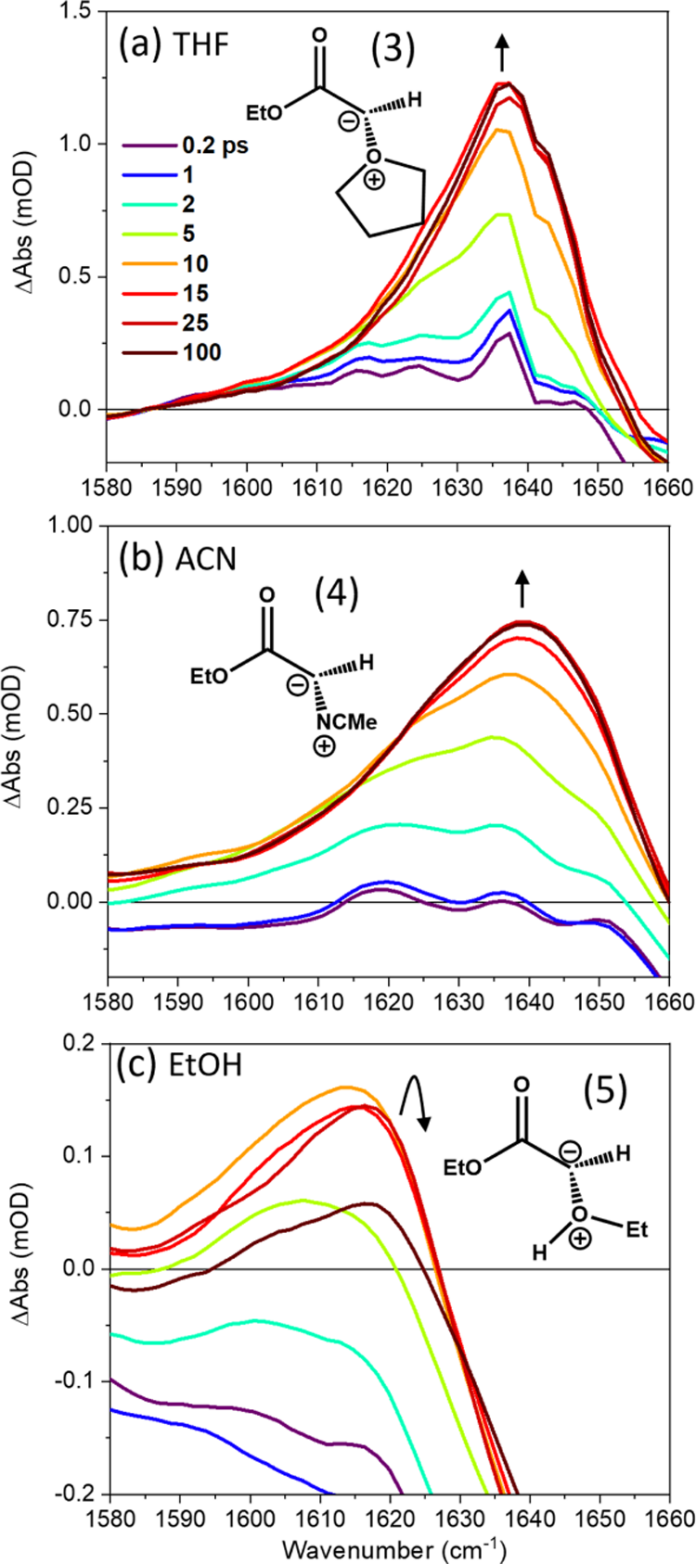
TRIR spectra spanning 1580–1660 cm^–1^ obtained for the 270 nm photoexcitation of 65 mM EDA in: (a) THF; (b) ACN; and (c) EtOH. The line colors indicate spectra obtained at different time delays shown by the inset key in panel (a). Black arrows show the directions of change in intensity of spectral features over time. The inset structures in panels (a)–(c) show the ylide products to which the transient absorption bands are assigned.

**Scheme 3:**
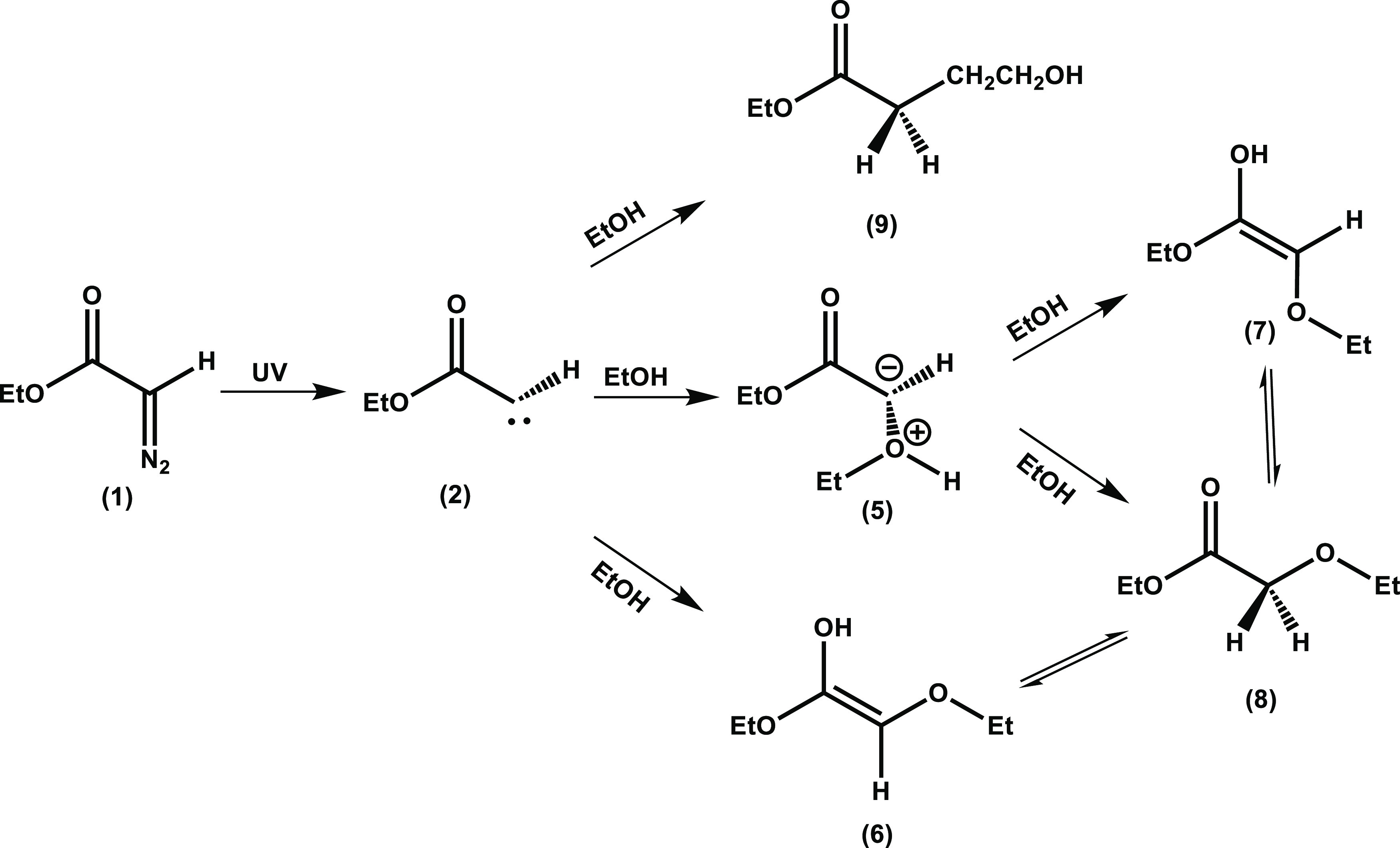
Production of EtOH-Y (5), Enol Intermediates (6) and (7), Ether (8), and C–H Insertion Product (9) from the Reaction of the Singlet Carbene (2) with Ethanol

The reaction of the singlet carbene (2) with nucleophilic solvents can either form α-carbonyl ylides (Scheme 1) or follow competing pathways such as C–H insertion reactions of the type shown in Scheme 2, for which TRIR data are presented in Section S1.1 of the SI. For the photolysis of EDA in EtOH, Scheme 3 summarizes the possible reaction pathways. The growth of ylide products following the 270 nm photoexcitation of EDA solutions was monitored using bands observed by TRIR at wavenumbers around 1600–1650 cm^–1^, examples of which are shown in Figure 1 for solutions in THF, ACN, and EtOH. Time-dependent integrated band intensities obtained from these spectra are plotted in Figure S2 of the SI, with Table 1 reporting the time constants for ylide production derived from exponential fits. Experiments conducted in cyclohexane show no evidence of the types of bands assigned here to ylides (see Figure S7 of the SI). The corresponding TRIR and band intensity data for an EDA/MeOH solution are shown in Figure S3 of the SI.

The features observed in the TRIR spectra in Figure 1 for the 270 nm photoexcitation of EDA in THF, ACN, and EtOH are consistent with a previous report by Xue et al.^54^ They show the formation of a THF-ylide (THF-Y) (3), with a characteristic absorption band at 1636 cm^–1^, an ACN-ylide (ACN-Y (4), 1642 cm^–1^), and an EtOH-ylide (EtOH-Y (5), 1617 cm^–1^). Computed vibrational frequencies for the carbonyl stretching modes of THF-Y (1642 cm^–1^), and ACN-Y (1643 cm^–1^) are in good agreement with the observed band positions. These calculations were for anti-isomers of the ylides, but both syn- and anti-isomers will contribute to the experimental band intensities for THF- and ACN-derived ylides. In contrast, we expect to observe mostly the anti-isomer of the EtOH-derived ylide because of the competing enol-forming pathway (see Section S1.2 of the SI). The THF-Y, ACN-Y, and EtOH-Y are formed with excess internal energy, as is evident from the broadening of the bands at earlier time delays and the narrowing and shifting of the bands to a higher wavenumber as this excess energy is transferred to the solvent bath. Reaction product bands were therefore decomposed by fitting to Gaussian functions with variable widths and centers to separate the vibrational cooling dynamics from population contributions to the band intensities and shapes. This spectral decomposition, which is illustrated in Figure S10 of the SI, also included partial recovery within the first 15 ps of an adjacent GSB feature located to a higher wavenumber in the TRIR spectra that is attributed to UV photoexcitation of the EDA.

The THF-Y, ACN-Y, and EtOH-Y bands grow with respective time constants of 6.5 ± 0.3, 8.4 ± 0.3, and 6.1 ± 0.4 ps (Table 1). The magnitudes of the time constants for the growth of these absorption bands in all three solvents suggest a rapid reaction of the singlet carbene with a molecule in its first solvent shell. The bands assigned to the carbonyl stretching motion of the EtOH-Y intermediates further decay with a time constant of 121 ± 10 ps, matching the time constant for the growth of a new band at 1745 cm^–1^. The 1745 cm^–1^ band is assigned to the ether product of EtOH-Y (5) rearrangements on the basis of steady-state irradiation experiments and alcohol concentration-dependent data reported by Xue et al. for Methanol-d_1_ and *t*-Butanol in ACN mixtures.^54^Section 3.2 provides further discussion of this ylide-to-ether transformation. The intensities and center wavenumbers of bands assigned to the THF-Y and ACN-Y species remain unchanged over the 1.3 ns time window probed.

In contrast to Xue et al., who found that the MeOD-Y band intensity grew with a first-order dependence on the Methanol-d_1_ concentration in ACN mixtures,^54^ we found no significant changes to the growth of the ylide photoproducts for measurements made in EtOH/THF, Cyclohexane/THF, and ACN/THF mixtures of different ratios (see Section 3.3, and Figures S18 and S19 of the SI). This finding is consistent with our interpretation that the carbene molecules formed from EDA photolysis react promptly with solvent molecules in their first solvent shell. Because Xue et al. performed their study in MeOD/ACN mixtures,^54^ we suggest an absorption band associated with solvation of the ACN-Y by MeOD overlaps the absorption by MeOD-Y at 1616 cm^–1^. This suggestion is supported by our observation in an EtOH/ACN mixed solution of a broad band centered around 1610 cm^–1^, which develops after the initial growth of ACN-Y and EtOH-Y bands (see Figure S9 of the SI). The 1610 cm^–1^ band does not decay in intensity over the course of our experiment, whereas we expect the EtOH-Y to decay completely within a few hundred picoseconds because of isomerization to an ether and enol (discussed below); hence we assign this overlapping band to a hydrogen-bonded complex of the ACN-Y.

### 3.2 Enol and Ether Formation

We recently reported the preferential formation of enol intermediates over ylides or carbocations during the reaction of an ethyl diazoacetoacetate-derived α-dicarbonyl carbene with alcohols.^55^ In the current work, we found evidence of a similar reaction pathway for the EDA-derived singlet α-carbonyl carbene, as is reported in Section 1.2 of the SI, with our findings summarized in Scheme 3. Here, we focus on the solvent-mediated proton transfer reaction of the EtOH-Y to form enol (7) and ether (8). Kinetic fits to the experimental data can be found in Section S4 and Figures S13–S17 of the SI.

**Figure 2:**
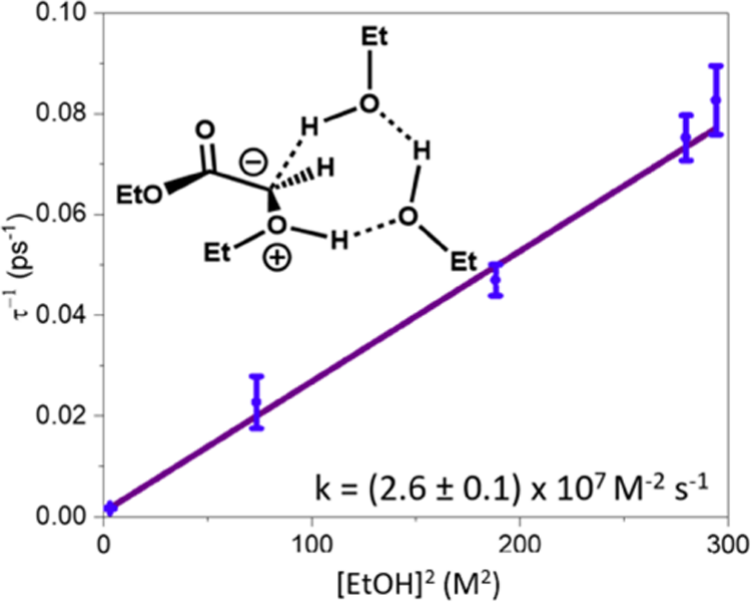
Quadratic dependence on the concentration of EtOH for the pseudo–first-order rate coefficients (τ^–1^) obtained for the growth of the ether product following photoexcitation of EDA in neat EtOH and various EtOH/THF mixtures. Error bars on the experimental data points (blue circles) show the uncertainties from the kinetic fits. A linear best fit line is shown in purple. The third-order rate constant, k, obtained from the gradient of the best fit line is shown in the inset. The structure shows a proposed reaction pathway involving a chain of two EtOH molecules.

The solvent-mediated proton transfer reaction of the EtOH-Y to form enol (7) and ether (8) can be tracked in EtOH/THF solutions by monitoring the TRIR bands at 1617 cm^–1^ (assigned to EtOH-Y, (5)), 1745 cm^–1^ (assigned to the ether, (8)) and 1722 cm^–1^ (assigned to the enol, (7)). Similar experiments were performed by Xue et al., who monitored the decay of the corresponding MeOD-Y band in MeOD/ACN mixtures and interpreted the kinetics as bimolecular (i.e., first order in [MeOD] and [MeOD-Y]).^54^ Because our results presented in Section 3.3 show the EtOH-Y band to be spectrally overlapped by a band for ethanol-solvated THF-Y, we preferred to track this rearrangement by monitoring the growth of ether and enol bands as a function of the EtOH concentration. Figure 2 summarizes the resulting kinetic data obtained for the formation of the ether. Figures S13–S17 of the SI show that the enol band intensity grows with the same time constant as that for the ether for each concentration of EtOH used. In contrast to Xue et al., we find the enol and ether bands grow with a quadratic dependence on the EtOH concentration, which suggests two EtOH molecules are required for the transformation. We speculate that proton transfer occurs along a chain of two EtOH molecules, such as the structure shown in the inset in Figure 2.

### 3.3 Ylide Solvation Dynamics in Binary Solvent Mixtures

**Figure 3:**
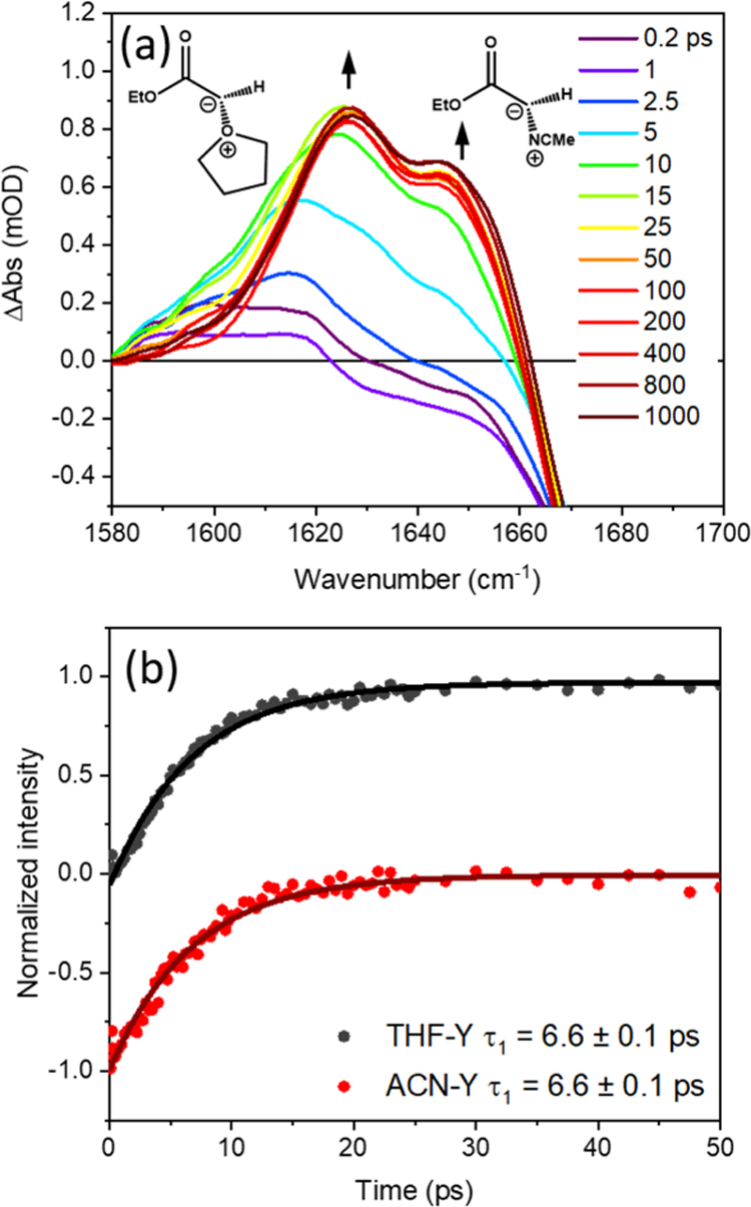
Effects of mixed THF and ACN solvents on ylide formation. (a) TRIR spectra obtained at wavenumbers from 1580–1700 cm^–1^ for the 270 nm photoexcitation of 65 mM EDA in a mixed solvent comprising 2:3 molar ratios of ACN/THF. The line colors indicate spectra obtained at different time delays shown by the inset key. Black arrows show the directions of change in intensity of the observed bands, and the structures identify band assignments to THF-Y and ACN-Y species. (b) Time-dependence of integrated band intensities (filled circles), normalized to maximum values of 1.0, and global kinetic fitting (solid lines) to single exponential functions for the THF-Y (black) and ACN-Y (red) ylides with structures shown in panel (a). The red data points and fit have been vertically offset by −1.0 for clarity.

The dynamics of THF-Y formation and solvation were further studied by dilution of the THF using either aprotic or protic solvents. Figure 3 shows TRIR spectra obtained in a mixed solution of ≈2:3 molar ratios of ACN/THF. Both THF-Y and ACN-Y ylides form in direct competition, as shown by bands observed at 1625 and 1651 cm^–1^, which grow with a common 6.6 ± 0.1 ps time constant (although this value may incorporate a component of vibrational cooling of the initially internally excited ylides). The ylide bands persist over longer time durations than the 1.3 ns experimental limit, with no changes to the band intensities or positions for time delays >25 ps, and therefore show no evidence for ylide exchange in this solvent mixture. The center peak positions of the THF-Y band in various ACN/THF mixtures (for which spectra are found in Figure S8 of the SI) are at a lower wavenumber than in the neat-THF solvent, and the difference in peak center position becomes larger for higher concentrations of ACN. Because these shifts in band positions are not time-dependent, they are likely to be due to the change in polarity of the solvent bath upon addition of ACN. This interpretation is supported by computed vibrational frequencies of the THF-Y with a PCM treatment of a neat ACN solvent bath, which predict a lower carbonyl stretching frequency (1625 cm^–1^) than for a PCM treatment of a THF solvent bath (1642 cm^–1^).

**Figure 4:**
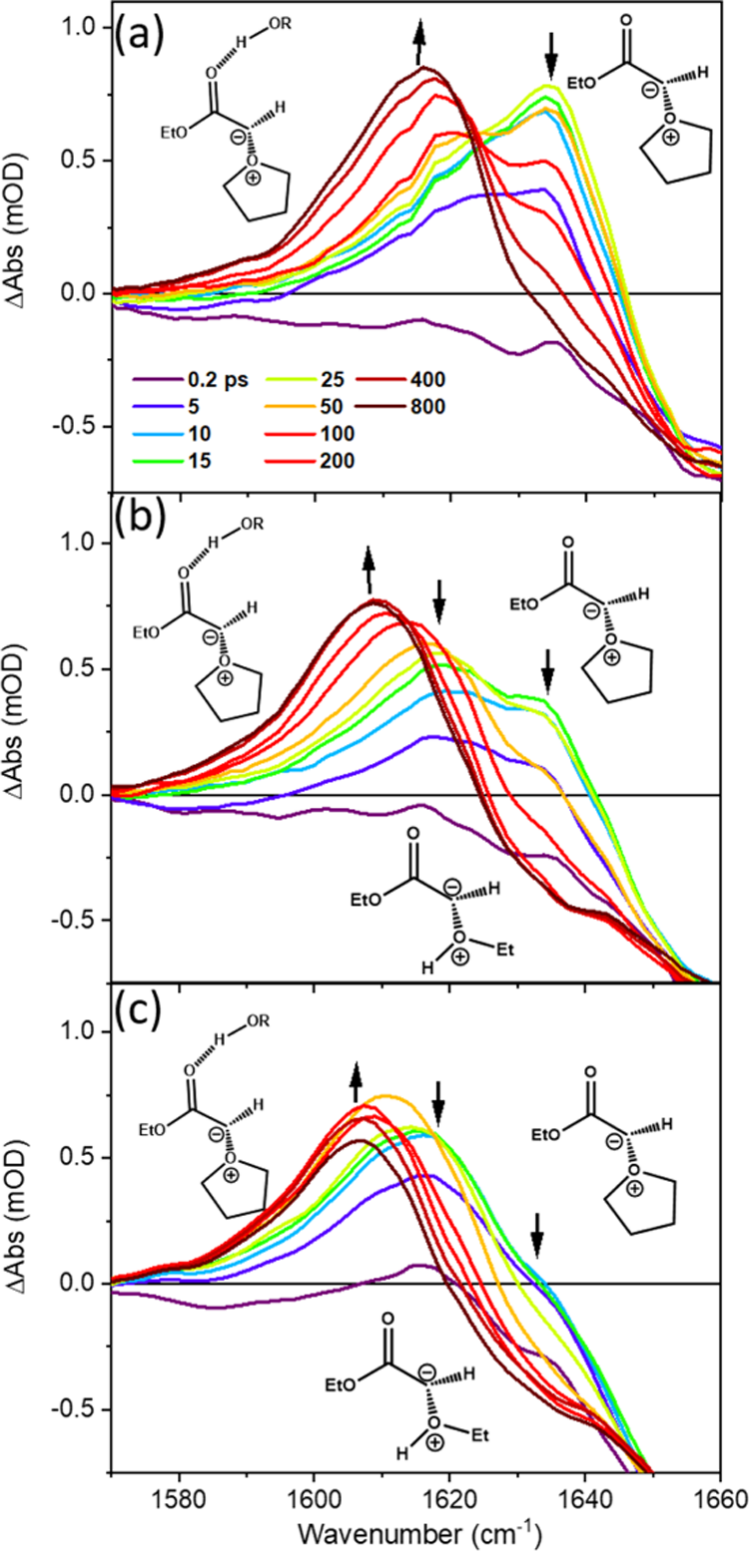
TRIR spectra measured in the wavenumber range 1570–1660 cm^–1^ for the 270 nm photoexcitation of 65 mM EDA in mixed EtOH/THF solutions of different ratios: (a) 0.4 M EtOH; (b) 1.7 M EtOH; and (c) 3.4 M EtOH. The line colors indicate spectra obtained at different time delays shown by the inset key in panel (a). Black arrows show the directions of change of spectral intensity for time delays after the initial growth of ylide bands is complete (∼25 ps).

In contrast, the ultrafast solvation dynamics of THF-Y in protic solvents are revealed by TRIR spectra obtained for EtOH/THF mixtures. Figure 4 shows example TRIR spectra in the 1570–1660 cm^–1^ wavenumber window measured for different EtOH/THF ratios. For EtOH concentrations above 1.7 M, three partially overlapping bands are identified between 1600 and 1650 cm^–1^, with centers at 1636, 1621, and 1608 cm^–1^. At a lower EtOH concentration of 0.4 M, only bands centered at 1636 and 1617 cm^–1^ are observed. At any given EtOH concentration above 1.7 M, the 1621 and 1636 cm^–1^ bands initially form with the same time constants but reveal further dynamics at later times, which will be the focus of the discussion here.

We assign the bands at 1636 and 1621 cm^–1^ in Figure 4 to the THF-Y and EtOH-Y ylides because of their close correspondence with the THF-Y band shown in Figure 1c and the EtOH-Y band shown in Figure 1d, as well as the dependence of the maximum band intensities on THF and EtOH concentration. These assignments show the competitive formation of both THF and EtOH solvent-derived ylides in the mixed EtOH/THF solvents. The EtOH-Y band decays by rearrangement to an enol or ether (Scheme 3) for time delays >25 ps, but the overlap by the wings of the bands at 1636 and 1608 cm^–1^ prevents an accurate kinetic analysis. We expect the EtOH-Y band to decay with the same time constant as the growth of the ether band at 1745 cm^–1^ (see Section 3.2).

**Scheme 4:**
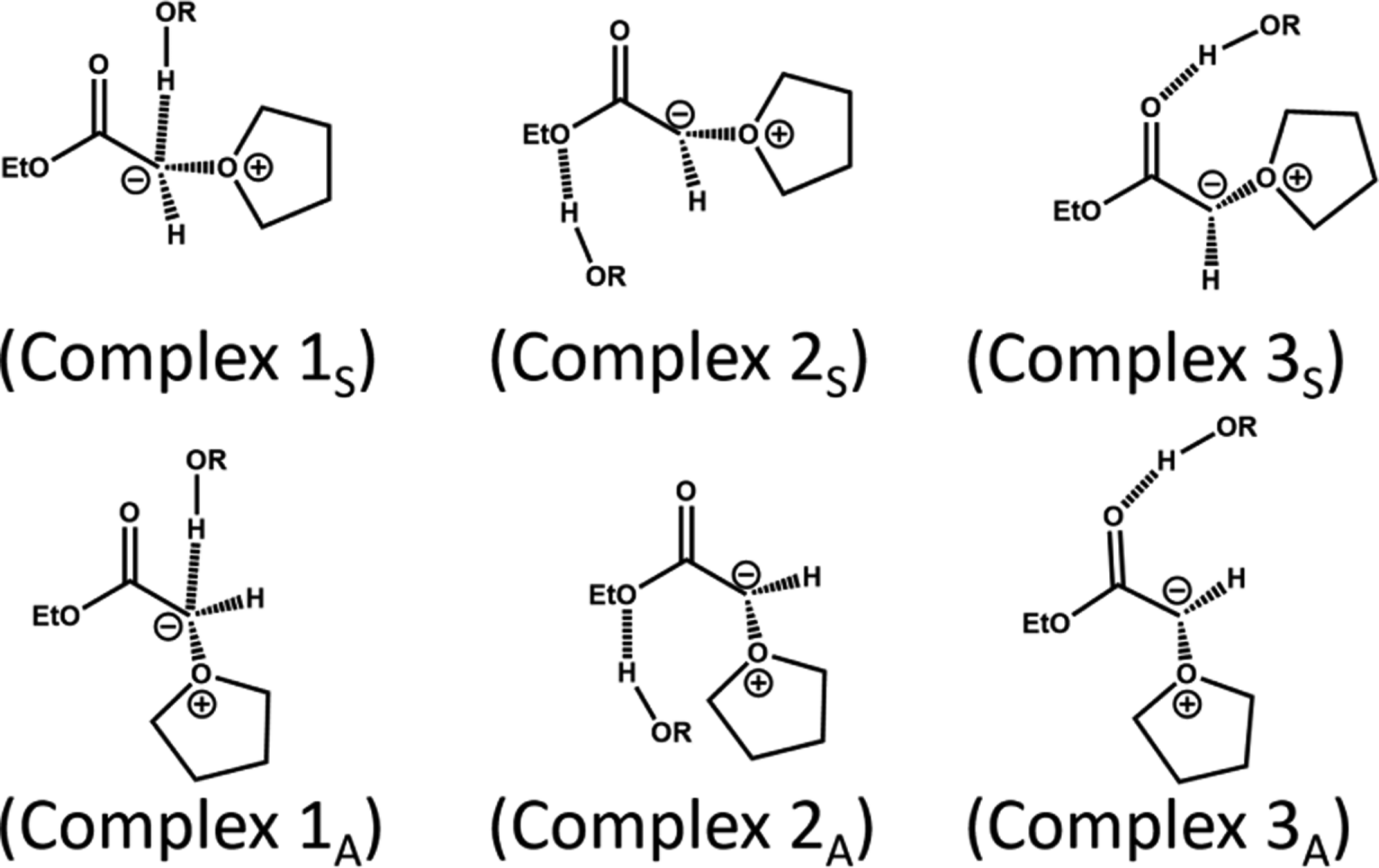
Schematic Structures Representing Computational Predictions for Possible Hydrogen-Bond Donor–Acceptor Complexes of Methanol and the Syn (S) or Anti (A) Isomers of THF-Y In the calculations for each structure, R = Me, but R = Et was used in experiments.

A kinetic analysis summarized in Figures S18 and S19 shows that the bands at 1617 cm^–1^ (in 0.4 M EtOH) and 1608 cm^–1^ (in >1.7 M EtOH) grow with the same time constant as the decay of the THF-Y bands. The reciprocal of the τ_2_ time constant for this growth of the 1617 and 1608 cm^–1^ bands depends linearly on the concentration of EtOH. We assign both the 1617 cm^–1^ (in 0.4 M EtOH) and 1608 cm^–1^ (in >1.7 M EtOH) bands to an IR signature for a hydrogen-bonded complex of the THF-Y with EtOH (referred to hereafter as a THF-Y HB complex). This assignment is supported by the observation in steady-state FTIR spectra (Figures S5 and S6 of the SI) of the formation of a hydrogen-bonded complex of ground-state EDA with EtOH, characterized by a 19 cm^–1^ downward shift in the carbonyl vibrational frequency relative to free syn-EDA. The photoexcitation of hydrogen-bonded EDA will likely result in a faster component for the formation of the THF-Y HB complex because EtOH molecules will already be close to THF-Y molecules. However, this component cannot be revealed by a kinetic analysis at earlier time durations because the THF-Y HB complex absorption band is overlapped by absorption from vibrationally hot THF-Y molecules. The various candidate structures considered for assignment of the hydrogen-bonded complex of the THF-Y with EtOH are shown in Scheme 4 and are further discussed in Section 3.4.

**Scheme 5:**
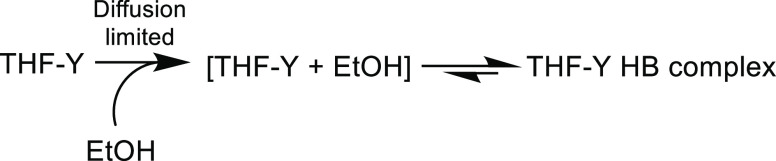
Complexation of THF-Y with EtOH in a EtOH/THF Solution

Scheme 5 shows a suggested mechanism for THF-Y solvation by EtOH in a EtOH/THF solution. The extent of decay of the uncomplexed THF-Y band (Figures 4 and S18a and S19) shows a preference for the THF-Y to exist in its EtOH-complexed form. Any thermal dissociation of the THF-Y HB complex will initially produce the hydrogen-bond donor and THF-Y confined as a pair within a solvent cage, as illustrated by [THF-Y + EtOH] in Scheme 5, from which the geminate pair can rapidly reform the HB complex. This interpretation is supported by the pseudo–first-order kinetic analysis of the formation of THF-Y HB complexes shown in Figure S18b of the SI, which yields a bimolecular rate coefficient *k* = (3.8 ± 0.4) × 10^9^ M^–1^ s^–1^ approaching the diffusion limit.

### 3.4 Structure of the THF-Y Hydrogen-Bonded Complex

To gain a deeper understanding of the hydrogen-bonded structure of the THF-Y complex with ethanol, identified by IR signatures at 1617 cm^–1^ (<0.4 M EtOH) and 1608 cm^–1^ (>1.7 M EtOH) (Figure 4), we computed various properties for the anti-isomer of each candidate structure shown in Scheme 4. Summaries are presented in Tables 2 and 3 and are used to guide our assignment of the preferred structure of the THF-Y HB complex described in Section 3.3. The calculations of the properties reported in Tables 2 and 3 used methanol as a protic solvent in place of EtOH to reduce the computational expense, but they are expected to inform about similar intermolecular interactions in solutions containing EtOH.

**Table 2:** Computed Enthalpies of Complexation, Δ_c_H, for the Various Hydrogen-Bonded Structures Illustrated in Scheme 4 for the Anti-Isomer of THF-Y

complex	Δ_c_H^MP2^ (kJ mol^–1^)^a^	Δ_c_H^B3LYP^ (kJ mol^–1^)^b^	Δ_c_H^B3LYP–D3^ (kJ mol^–1^)^c^
1_A_	–41.4	–24.9	–39.9
2_A_	–31.0	–17.8	–31.8
3_A_	–39.9	–30.7	–39.1

a Computed using MP2/6-311++G(d,p) geometries and MP2/Aug-cc-pVTZ single point energies.

b Computed using B3LYP/6-31+G(d) geometries and single point energies.

c Computed using B3LYP-D3/6-31+G(d) geometries and single point energies.

**Table 3:** Computed Hydrogen-Bond Lifetimes and Vibrational Frequencies for the Various Hydrogen-Bonded Structures Illustrated in Scheme 4 for the Anti-Isomer of THF-Y

complex	median hydrogen-bond lifetime^a^ (ps)	median hydrogen-bond lifetime^b^ (ps)	computed carbonyl vibrational wavenumber^c^ (cm^–1^)
free THF-Y_A_			1642
1_A_	0.30	0.30	1649
2_A_	0.61	1.4	1657
3_A_	2.6	3.6	1623

a Computed at the B3LYP/6-31+G(d) level of theory.

b Computed at the B3LYP-D3/6-31+G(d) level of theory.

c Computed at the MP2/6-311++G(d,p) level of theory.

**Figure 5:**
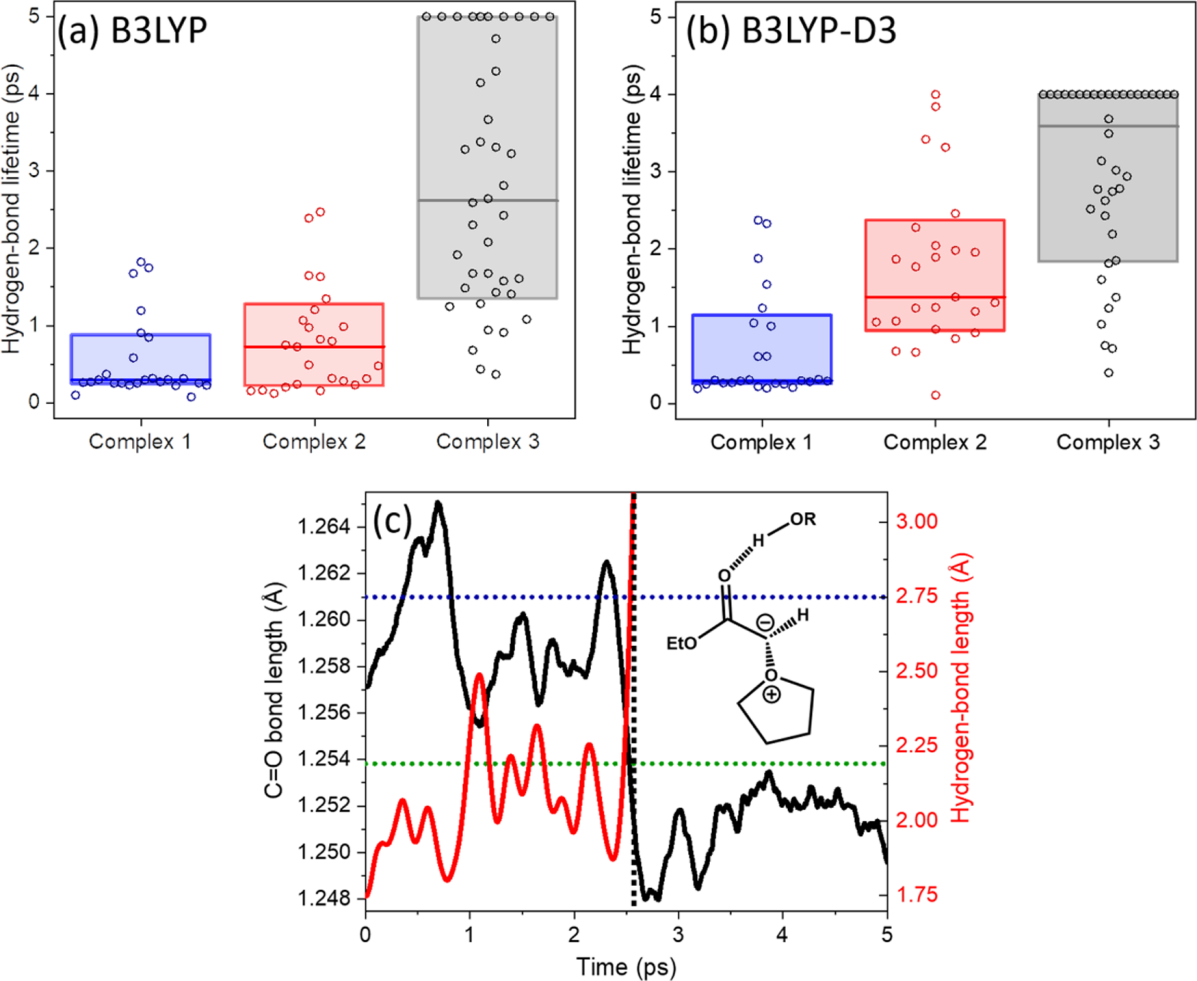
Trajectory simulation outcomes for anti-isomers of the THF-Y complexes with methanol at 300 K. (a) Hydrogen-bond lifetimes obtained from multiple trajectories computed at the B3LYP/6-31+G(d) level of theory and initiated from complexes 1_A_, 2_A_, and 3_A_. (b) Corresponding lifetimes for trajectories computed at the B3LYP-D3/6-31+G(d) level of theory. Box plots show the mean hydrogen-bond lifetimes (center horizontal lines) and the middle 80 percentile (box length). (c) Evolution of C=O and H···O (shown as the dashed line in the inset structure) internuclear distances during a representative trajectory for dissociation of complex 3_A_, computed at the B3LYP level of theory. The time-dependent variation of the C=O bond length is shown in black, and the hydrogen-bond length is shown in red, with both smoothed by a 0.25 ps moving average. Horizontal dotted lines show computed C=O bond lengths for complex 3_A_ of THF-Y with methanol (blue) and for uncomplexed THF-Y (green).

Computational values for the enthalpy of complexation shown in Table 2 indicate that all three complexes experience thermodynamic stabilization compared to the uncomplexed THF-Y at the various levels of theory used. To gain deeper insights into the kinetic stability of the complexes, dynamical simulations were also performed, with the results summarized in Table 3. ADMP trajectories were propagated at the B3LYP and B3LYP-D3 levels of theory, using the 6-31+G(d) basis set and starting from complexes 1_A_, 2_A_, or 3_A_. The trajectory propagations used a constant temperature (300 K) and continued until the hydrogen bond length exceeded 3.1 Å, or for a maximum duration of either 5 ps at the B3LYP level of theory or 4 ps at the B3LYP-D3 level of theory. A total of 25 trajectories were computed for each of complexes 1_A_ and 2_A_, and 40 for the 3_A_ isomer, using both the B3LYP and B3LYP-D3 methods, all with randomized starting conditions. The statistics of computed lifetimes for the complexes are shown in Figure 5, together with a representative trajectory.

Of the three starting structures, Complex 3_A_ shows enhanced kinetic stability compared to complexes 1_A_ and 2_A_ for trajectories propagated at both B3LYP and B3LYP-D3 levels of theory, with median THF-Y HB lifetimes reported in Table 3. Of the B3LYP trajectories computed from a starting structure corresponding to Complex 3_A_, 9 out of 40 showed a hydrogen-bond lifetime exceeding 5 ps. The same analysis for the B3LYP-D3 trajectories computed from a starting structure corresponding to Complex 3_A_ shows that 19 out of 40 resulted in a hydrogen-bond lifetime exceeding 4 ps. In contrast, for trajectories initiated from Complex 2_A_, only 1 out of 25 gave a hydrogen-bond lifetime exceeding 4 ps. The larger median hydrogen-bond lifetimes computed for complexes 2_A_ and 3_A_ using the B3LYP-D3 methodology compared to B3LYP suggest a contribution from dispersion forces to the stability of the hydrogen-bonded complexes, but a greater number of trajectories is required for a statistically robust analysis. Overall, the calculated enthalpies of complexation, the simulated complex lifetimes, and computed carbonyl stretching frequencies suggest a greater propensity for the formation of Complex 3 in a solution at dynamic equilibrium.

The experiments observe the change in stretching frequency of the THF-Y carbonyl group, and the results from the trajectories confirm the idea that the hydrogen-bonding environment is connected to changes in carbonyl bond length and hence, vibrational frequency. The calculations therefore support our interpretation that the complexed and free forms of the carbonyl group in THF-Y are spectroscopically distinguishable species. Moreover, the trajectories indicate that the solvent interactions with THF-Y undergo spontaneous fluctuations on timescales of a few picoseconds. The computed evolutions of C=O bond and hydrogen-bond lengths are illustrated in Figure 5c for one trajectory corresponding to the thermal breakup of complex 3_A_, which shows a significant shift in the average C=O bond length at a time delay of about 2.5 ps, matching the time at which the hydrogen-bond length exceeds 3.1 Å. The time at which these changes occur is taken to be the computed hydrogen-bond lifetime from this trajectory. The combined results from multiple trajectory simulations show the complexation to the carbonyl group to be the most probable H-bonding site of the three options considered. The computed vibrational frequency of complex 3_A_ (1623 cm^–1^) is in good agreement with the observed 1617 cm^–1^ band in Figure 4 and reproduces the −17 cm^–1^ shift from the uncomplexed THF-Y observed in experiments and summarized in Table 3.

We speculate that the shift in band position of the THF-Y complex with EtOH from 1617 to 1608 cm^–1^ for higher concentrations of EtOH could be a result of a microscopically heterogeneous distribution of EtOH in THF. More specifically, we suggest that the THF-Y preferentially forms within a locally THF-dominated environment, but it can then diffuse to a locally EtOH rich environment, where it forms complexes 3_S_ and 3_A_. This interpretation is supported by computed carbonyl stretching bands of complex 3_A_ at 1623 cm^–1^ in THF and 1614 cm^–1^ in EtOH (using MeOH as a substitute for EtOH in the computations and a PCM treatment of further EtOH solvent interactions). These calculations predict a shift of this band to a lower wavenumber in the alcohol solution of the same magnitude as the experimental observations, as shown in Table 3. We discount the assignment of the 1617 and 1608 cm^–1^ bands to complexes 1 and 2 because computed vibrational frequencies instead predict a shift in the carbonyl stretching band to a higher wavenumber for these specific forms of THF-Y interaction with an alcohol solvent molecule.

### 3.5 C–H Hydrogen Bonding to THF-Y

**Figure 6:**
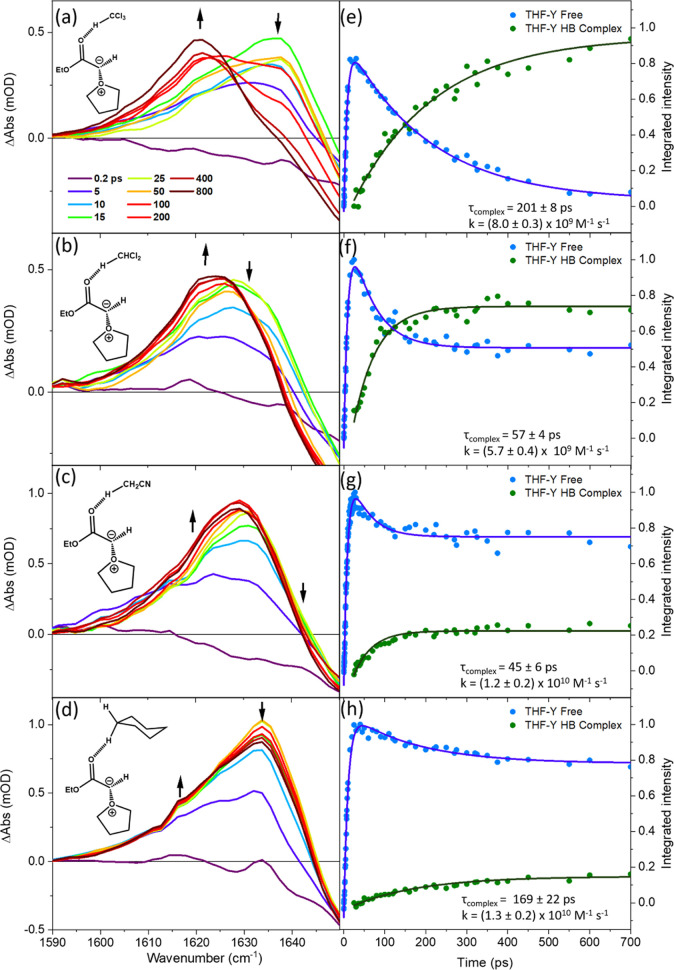
Evidence from TRIR spectra for C–H hydrogen-bond donation to THF-Y species in mixed solutions of THF and either CHCl_3_, CH_2_Cl_2_, ACN, or cyclohexane. Left column: TRIR spectra for the wavenumber range from 1590–1650 cm^–1^ for the photoexcitation of 65 mM EDA solutions of: (a) 0.62 M Chloroform in THF; (b) 3.1 M DCM in THF; (c) 1.9 M ACN in THF; and (d) 0.46 M Cyclohexane in THF. The line colors indicate spectra obtained at different time delays shown by the inset key in panel (a). Black arrows show the directions of change of the spectral intensity for delays longer than 25 ps. Right: Exponential fitting for the growth and decay of integrated IR band intensities assigned to THF-Y (blue), and for the growth of the solvent-complexed ylide (green) for EDA solutions of (e) 0.62 M Chloroform in THF; (f) 3.1 M DCM in THF; (g) 1.9 M ACN in THF; and (h) 0.46 M Cyclohexane in THF. Inset numbers report the exponential time constants and estimated bimolecular rate coefficients for solvent complexation. Data points collected at delays from 0–25 ps were omitted from the exponential fitting of the integrated intensities of the THF-Y HB complex band because of spectral overlap with the absorption from vibrational hot THF-Y molecules. Band centers reported in Table 4 were determined using the spectral decomposition method reported in Figures S11 and S12 of the SI.

The HB acceptor properties of the THF-Y were further explored using weaker HB donors than EtOH. Figure 6 shows the TRIR spectra obtained for Chloroform/THF, DCM/THF, ACN/THF, and Cyclohexane/THF mixtures. Example spectral decompositions for the ACN/THF and Cyclohexane/THF mixtures are presented in Figure S12 of the SI. The same spectral decomposition method was applied to Chloroform/THF and DCM/THF mixtures. The findings are summarized in Table 4. Taking the example of an ACN/THF mixture with 1.9 M ACN shown in Figure 6c, a partial decay of the THF-Y band centered at 1633 cm^–1^ occurs for time delays >25 ps, with a time constant that matches the growth of a weak new band at 1624 cm^–1^. The 1624 cm^–1^ band is not thought to be from ACN-Y because of the observed center wavenumber and the timescale for its growth. Electrostatic complexation is also unlikely to be responsible for the −9 cm^–1^ band shift because similar effects are observed in nonpolar solvents such as cyclohexane. Instead, the changes are suggested to arise through hydrogen bonding from the slightly polar C–H bonds of ACN to THF-Y. At ACN concentrations >1.9 M, the HB complexation is not observed, perhaps because intermolecular ACN interactions become more favorable than the weak hydrogen bonding to THF-Y. Corresponding assignments to THF-Y HB complexes account for the band shifts seen in the other solvents.

**Table 4:** TRIR Band Positions for THF-Y and its Complexes with Various Hydrogen-Bond Donors and a Lithium Salt

	THF-Y band (cm^–1^)	THF-Y HB complex band (cm^–1^)	shift (cm^–1^)
EtOH	1636	1617/1608^a^	–19/–28^a^
chloroform	1636	1622	–14
DCM	1631	1622	–10
ACN	1633	1624	–9
cyclohexane	1634	1625	–9
LiCl	1638	1622	–16

a At higher concentrations of EtOH (see main text).

Even for the solvents that are weaker hydrogen-bond donors than EtOH, the THF-Y HB complexes form with bimolecular rate coefficients consistent with diffusion-limited kinetics. The values of these rate coefficients are reported in Figure 6. The ylide HB complexes show a smaller shift in the carbonyl stretching frequency relative to the free THF-Y than observed in EtOH, with the smallest shifts found for the weakest hydrogen-bond donors (DCM, cyclohexane, and ACN).

The decay amplitude of the free THF-Y band informs about the equilibrium between free THF-Y and the THF-Y HB complex. Although the magnitude of this decay will depend on the concentration of the HB donor, which differs for the examples shown, the data suggest equilibration to the complexed ylide is more favorable in chloroform than in DCM, ACN, and cyclohexane. Again, a faster component to the growth of the THF-Y HB complex absorption bands in solutions with the C–H hydrogen-bond donors cannot be discounted because of overlapping absorption by the newly formed, vibrationally hot THF-Y molecules.

### 3.6 Ylide Complexation Dynamics with Li^+^ Ions

**Figure 7:**
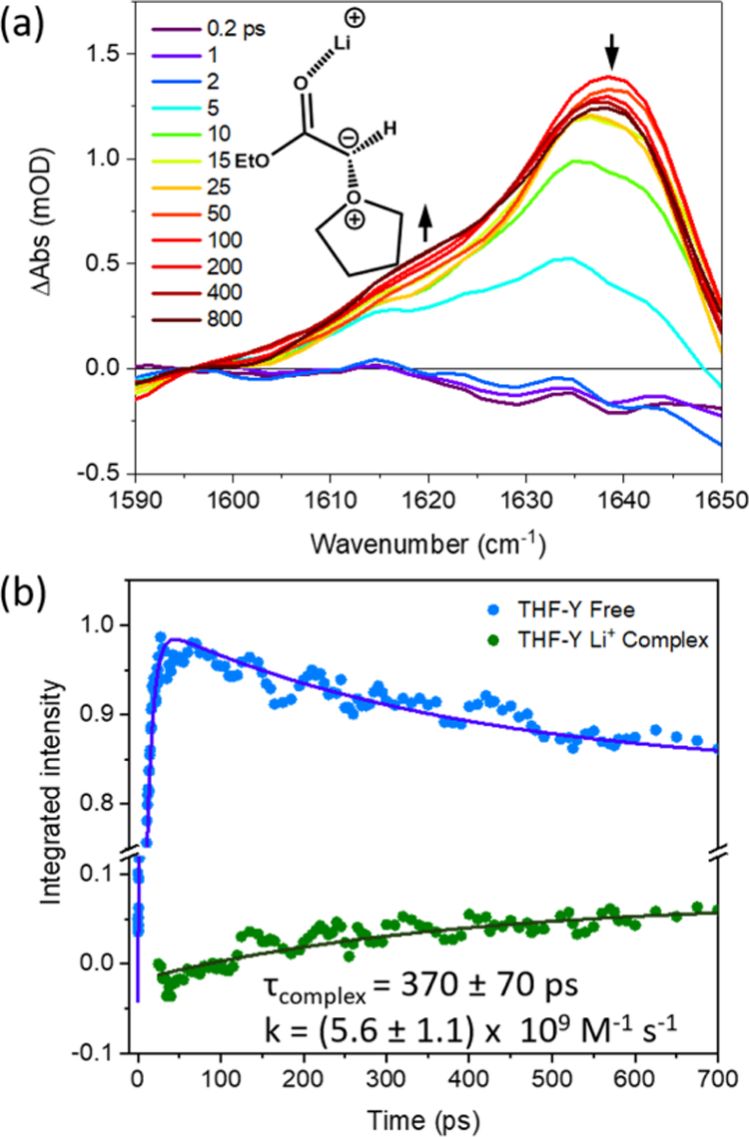
Evidence from TRIR spectra for THF-Y complexation with Li^+^. (a) TRIR spectra in the range 1590–1650 cm^–1^ obtained for the 270 nm photoexcitation of 65 mM EDA in THF with 0.47 M LiCl. The line colors indicate spectra obtained at different time delays shown by the inset key. The structure of the proposed complex is shown in the inset. (b) Exponential global fitting for the growth and decay of IR band intensities assigned to THF-Y (blue) and for the growth of the Li^+^-complexed ylide (green). Data points for time delays less than 25 ps for the THF-Y complex have been omitted because of overlapping absorption bands from vibrationally hot THF-Y molecules. Inset numbers report the exponential time constant and estimated bimolecular rate coefficient for complexation.

The complexation dynamics of THF-Y with Li^+^ ions are revealed by TRIR spectra obtained for solutions of EDA in THF with LiCl added to a concentration of 0.47 M, as exemplified in Figure 7. TRIR spectral decomposition was carried out using the same methods as described in Section 3.5. The time-dependent spectral shifts of the THF-Y species with the addition of LiCl are similar to those observed with the hydrogen-bond donors discussed in Sections 3.3–3.5, suggesting preferential coordination to the carbonyl group. For example, a band shifted by −16 cm^–1^ relative to the free THF-Y band grows at time delays >25 ps, and the time constant for its growth is consistent with a bimolecular rate coefficient close to the diffusion limit (Figure 7b). The observed spectral shift agrees well with the computed −19 cm^–1^ shift in the carbonyl stretching frequency of the THF-Y complexed to Li^+^. A calculated enthalpy of complexation suggests that the Li^+^ ion coordination stabilizes the THF-Y by −64 kJ mol^–1^. Despite this greater stabilization compared to calculations for MeOH, the observed position of equilibrium still favors the free THF-Y. We suggest that the majority of Li^+^ ions are preferentially solvated by THF molecules and are not available for complexation with the ylide. These findings are consistent to those of Reitz et al., who identified a hyperbolic relationship between the concentrations of LiBr and the E/Z-isomer product ratios in the Wittig reactions.^47^ They argued that only at higher concentrations of LiBr in THF were Li^+^ cations available for complexation with betaine intermediates.

### 3.7 Changes to the Electronic Structure of THF-Y in Complexes with Hydrogen-Bond Donors or Li^+^ Ions

**Scheme 6:**
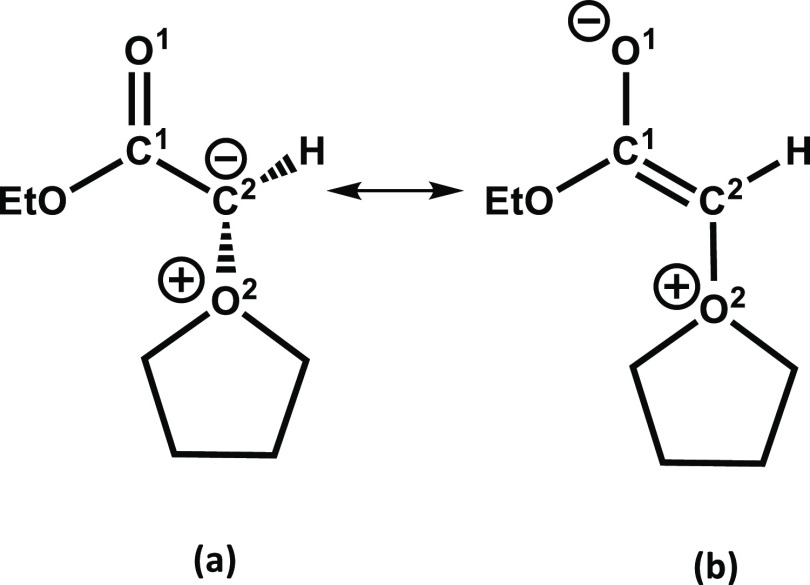
Resonance Structures of THF-Y, Highlighting the Stereochemistry around the C^2^ Atom

The shifts in carbonyl stretching frequency of the THF-Y complexed with the various hydrogen-bond donors or Li^+^ ions suggest changes to the molecular and electronic structure of the ylide induced by complexation. α-Carbonyl ylides can be described by two resonance structures, as exemplified for THF-Y in Scheme 6. Table 5 reports computed structural parameters for THF-Y and its complexes with MeOH and a Li^+^ ion, which guide the discussion in this section. The conjugation of the carbanion into the carbonyl group has previously been suggested to account for differences in reactivity of stabilized and nonstabilized ylides in Wittig reactions^28^ and therefore, understanding how solvation alters this conjugation should offer insights into the causes of solvent effects in ylide-mediated reactions.

**Table 5:** Computed Structural Properties of THF-Y and Its Complexes with MeOH or a Li^+^ Ion^a^

THF-Y	C^1^C^2^ (Å)	C^2^O^1^ (Å)	∠O^1^C^2^C^1^H (deg)	∠O^1^C^2^C^1^O^2^ (deg)
free	1.405	1.241	26.6	169.9
MeOH HB complex	1.389	1.254	9.8	174.0
Li^+^ ion complex	1.381	1.265	6.9	178.7

a Calculations used the MP2 level of theory with a 6-311++G(d,p) basis set.

The stereochemistry around the C^2^ atom is largely controlled by the resonance structures, as shown in Scheme 6 because of the different hybridization characters of the C^2^ atom in each resonance form. This stereochemistry can therefore inform about the electronic structure of THF-Y and its complexes. The dihedral angles computed for the free THF-Y are reported in Table 5 and deviate from expectations for both sp^2^ (0° with respect to O^1^C^1^C^2^H) and sp^3^ (60° with respect to O^1^C^1^C^2^H) carbons. The computed stereochemistry therefore suggests no preference for either resonance structure; instead, the negative charge is shared nearly equally between the C^2^ and O^1^ atoms. The O^1^C^1^C^2^O^2^ dihedral angle is smaller than the O^1^C^1^C^2^H dihedral angle, possibly because of the electrostatic interactions with the lone pair from the ester functional group. The O^1^C^1^C^2^H dihedral angle is likely to be a more reliable indicator of the C^2^ hybridization state because the H atom is less affected by steric and electrostatic interactions. Complexation of the THF-Y with MeOH or a Li^+^ ion causes the dihedral angles O^1^C^1^C^2^H and O^1^C^1^C^2^O^2^ to decrease, in addition to changes to the C^1^C^2^ and C^1^O^1^ bond lengths, which reduce and lengthen, respectively. These structural changes suggest hydrogen bonding or Li^+^ ion coordination increases the character of resonance form (b) in Scheme 6, in which the C^2^ atom is sp^2^-hybridized. For THF-Y complexed with Li^+^, resonance form (b) appears to dominate, with the negative charge mostly localized onto the O^1^ atom.

## 4 Conclusions

The UV photoexcitation of ethyl diazoacetate in solution and the resulting elimination of N_2_ forms singlet α-carbonyl carbene intermediates, which rapidly react with solvent molecules by insertion into C–H bonds. In nucleophilic solvents, the carbene also competitively reacts to produce ylides. In this study, these competing processes were resolved by ultrafast TRIR spectroscopy. The interaction of the THF-ylide, THF-Y, with EtOH molecules, was experimentally monitored in EtOH/THF solvent mixtures via the growth of a band at 1617 cm^–1^ (in 0.4 M EtOH) and 1608 cm^–1^ (>1.7 M EtOH). This spectral feature was assigned to the hydrogen-bonded complex of EtOH with THF-Y, with complexation showing nearly diffusion-limited kinetics. To characterize the hydrogen-bonding interaction further, atom-centered density matrix propagation trajectories were computed at 300 K for various complexes with MeOH. These dynamical simulations identified hydrogen-bond donation to the carbonyl group of the THF-Y to have greater kinetic stability than complexes in which the hydrogen-bond donation is to the other acceptor sites shown in Scheme 4. Transient IR bands that shift to a lower wavenumber than the uncomplexed THF-Y band also revealed that the THF-Y species interacts with chloroform, dichloromethane, or cyclohexane by accepting a C–H hydrogen bond, as well as with Li^+^ ions. The shifts in the band centers reduce in magnitude with weaker hydrogen-bond donors. The computed (MP2) structures of THF-Y and its complexes with MeOH and Li^+^ indicate that complexation induces a shift of negative charge into the carbonyl group of the ylide. The outcomes reveal the dynamical behavior of these elusive intermediates in a range of organic solvents commonly used in synthetic chemistry. They also provide new insights into how ylide–solvent and ylide-Li^+^ interactions change the sp^2^ hybridization character of the ylidic C atom in the studied α-carbonyl ylides which may influence the ylide-mediated reaction mechanisms. For example, such changes might play a role in determining the outcomes of competing [1,2] and [2,3]-sigmatropic rearrangements^40−42^ because greater sp^2^ character at the ylidic C atom of the reactant will shift the transition state for the [2,3]-rearrangement later along the reaction pathway. Biswas and Singleton showed how hydrogen bonding can exert control over this competition,^40^ but Li^+^-ion coordination might also be similarly effective. The changes in electronic character induced by ylide–solvent or ylide-Li^+^ interactions may also contribute to the selectivity of other reactions of α-carbonyl ylides, such as the Wittig olefination.^30,32,37^

## Supporting Information Available

Experimental data and analysis for carbene C–H insertion and enol and ether-forming reaction pathways, steady-state UV–vis spectra, steady-state FTIR spectra, TRIR spectra and spectral decomposition procedures, and computational results (PDF)

## Notes

Data are available at the University of Bristol data repository, data.bris, at https://doi.org/10.5523/bris.38zbrp39vx4az2dmvyzm1440ud.
