# Supplementary material for: Direct Observation of the Dynamics of Ylide Solvation by Hydrogen-bond Donors Using Time-Resolved Infrared Spectroscopy
Source: J Am Chem Soc. 2022 May 17;144(21):9330–43. doi: 10.1021/jacs.2c01208 (PMC9164226; doi:10.1021/jacs.2c01208)
Supplement: Supplementary file 1 — ja2c01208_si_001.pdf [file ja2c01208_si_001.pdf]

Supporting Information for "**Direct Observation of the Dynamics of Ylide Solvation by Hydrogen-bond Donors using Time-Resolved Infrared Spectroscopy**"

Ryan Phelps* and Andrew J. Orr-Ewing*

School of Chemistry, University of Bristol, Cantock's Close, Bristol BS8 1TS, UK.

* Authors for correspondence. E-mail: ryan.phelps@ed.ac.uk, a.orr-ewing@bristol.ac.uk

Contents

Section S1 – Alternative Carbene Reaction Pathways

Figure S1 – TRIR spectra spanning 1725 – 1780 cm^{-1} obtained for the 270-nm photoexcitation of 65 mM solutions of EDA in: (a) Cyclohexane; (b) THF; (c) ACN; (d) EtOH. 4

Figure S2 - Time-dependent integrated band intensities and kinetic fits for C-H insertion products and solvent derived ylides following the 270-nm photoexcitation of 65 mM EDA in: (a) Cyclohexane; (b) THF; (c) ACN; (d) EtOH. 5

Figure S3 - TRIR spectra for the 270-nm photoexcitation of 65 mM EDA in methanol, in the wavenumber ranges (a) 1580-1660 cm^{-1} and (b) 1705-1780 cm^{-1}. 7

Scheme S1 – Production of E and Z Enol isomers, MeOH-Y, and an Ether from the reaction of the singlet carbene with methanol....8

Scheme S2 – Proposed mechanism for the concerted addition of MeOH to carbene (2) to form the E-isomer of the Enol....8

Section S2 – Supporting FTIR, UV-Vis and Time-Resolved Infrared Spectra

Figure S4 – UV-Vis spectra measured in the 255-280 nm range for 65mM EDA solutions 9

Figure S5 – FTIR spectra measured in the wavenumber range 1640-1800 cm^{-1} (Left) and 2060-2160 cm^{-1} (Right) for 65 mM EDA solutions. 9

Figure S6 – Left - FTIR spectra measured in the wavenumber range 1640-1740 cm^{-1} for 65 mM EDA solutions with various concentrations of EtOH in CycH. Right – Hydrogen-bonded EDA : uncomplexed EDA band intensity ratio dependence on EtOH concentration. 10

Figure S7 - TRIR spectra obtained at wavenumbers from 1550-1780 cm^{-1} for the photoexcitation of a 65 mM solution of EDA in cyclohexane. 10

Figure S8 – TRIR spectra obtained in the range 1580-1670 cm^{-1} for the photoexcitation of a 65 mM solution of EDA in mixed solvents of THF:ACN with the specified ratios: Top - 9:1; Middle - 8:2; Bottom - 6:4. 11

Figure S9 – Left - TRIR spectra obtained at wavenumbers from 1570-1660 cm^{-1} for the photoexcitation of a 65 mM solution of EDA in 9.6M EtOH in ACN. Right – Kinetic fitting of integrated band intensities of the EtOH-Y, ACN-Y, and the ACN-Y HB complex. 12

Section S3 – TRIR Spectral Decomposition

Figure S10 – Example fitting procedure for the decomposition of overlapping bands correpsonding to THF-Y, C-H insertion products, and and the ground state bleach (GSB), observed following the 270-nm photoexcitation of 65 mM EDA in THF. 14

Figure S11 – Example fitting procedure for the decomposition of overlapping EtOH-Y, THF-Y, and THF-Y HB complex bands for the 270-nm photoexcitation of 65 mM EDA with 1.7 M EtOH in THF. 15

Figure S12 – Example fitting procedure for the decomposition of overlapping THF-Y and THF-Y HB complex bands for the 270-nm photoexcitation of 65 mM EDA with 1.9 M ACN in THF (Left) and 0.46 M Cyclohexane in THF (Right). 16

Section S4 – Supporting Kinetic Fits

Figure S13 – Time-dependence of integrated band intensities and kinetic fits for a 17.2 M (neat) solution of EtOH. (a) Enol (b) Ether (c) EtOH-Y. 17

Figure S14 – Time-dependence of integrated band intensities and kinetic fits for a 16.7 M solution of EtOH in THF. (a) Enol (b) Ether. 18

Figure S15 – Time-dependence of integrated band intensities and kinetic fits for a 13.7 M solution of EtOH in THF. (a) Enol (b) Ether. 18

Figure S16 – Time-dependence of integrated band intensities and kinetic fits for an 8.6 M solution of EtOH in THF. (a) Enol (b) Ether. 19

Figure S17 – Time-dependence of the Ether integrated band intensity and the kinetic fit for a 1.7 M solution of EtOH in THF. 19

Figure S18 – Kinetics of photoproduct formation after 270-nm photoexcitation of 65 mM EDA in a mixed solution of 3.4 M EtOH in THF. (a) Integrated intensities of TRIR bands assigned to the THF-Y and THF-Y HB complex, and biexponential fits. (b) Dependence on the concentration of EtOH of the pseudo-first order rate coefficients ($1/\tau_2$) for the growth of the THF-Y HB complex....................20

Figure S19 - Time-dependence of integrated band intensities and kinetic fits for THF-Y and the THF-Y HB complex for EtOH:THF solvent mixtures....................21

Section S5 – Computational

Table S1 – Computed and Observed Vibrational Wavenumbers of EDA Isomers in Various Solvents.......22

Table S2 – Computed Vibrational Wavenumbers of Carbene Intermediates....................23

Table S3 – Reported Vibrational Wavenumbers from Prior End-Product Analysis and Observed Vibrational Wavenumbers from the TRIR Measurements in the Current Work for C-H Insertion Products Formed in Various Solvents. 23

Table S4 – Computed and Observed Vibrational Wavenumbers of Ylide and Enol Intermediates Formed in Various Solvents. 24

Table S5 – Computed and Observed Vibrational Wavenumbers of Hydrogen-Bonded Complexes of THF-Y with Various Hydrogen-Bond Donors....................24

Section S1 – Alternative Carbene Reaction Pathways

In competition with the ylide-forming pathways that are the focus of the main manuscript, the carbenes produced from UV photolysis of ethyl diazoacetate (EDA) can insert into C-H bonds of various organic solvent molecules, or they can react with alcohols to make enols and ethers. The spectroscopic evidence for these competing pathways, and the derived kinetics, are reported here.

S1.1 C-H bond insertion

Evidence for C-H bond insertion comes from data such as those in Figure S1, which shows representative TRIR spectra spanning 1725 - 1780 cm^{-1} obtained following the 270-nm photoexcitation of 65 mM solutions of EDA in cyclohexane, THF, ACN and EtOH. Kinetic analyses of the integrated band intensities for C-H insertion products are compared with those for the competing ylide intermediate pathways (see Section 3.1 of the main manuscript) in Figure S2.

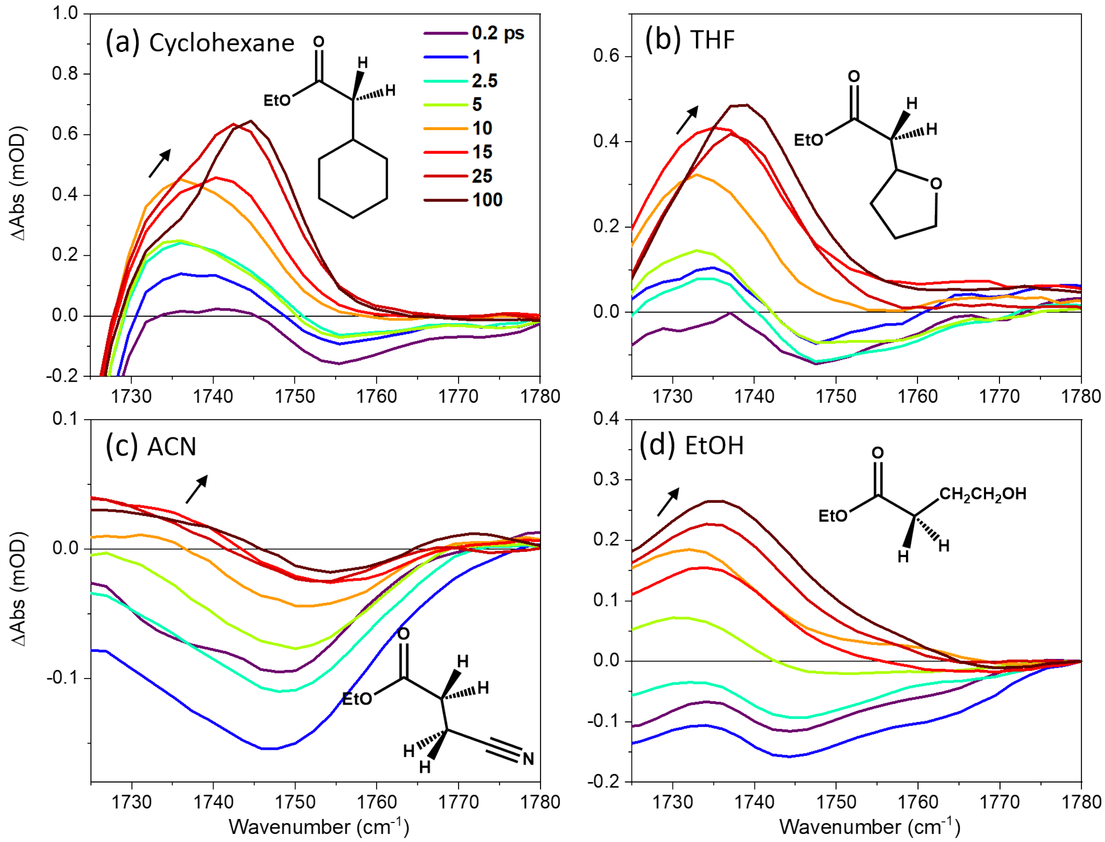

Figure S1 – TRIR spectra spanning 1725 – 1780 cm^{-1} obtained for the 270-nm photoexcitation of 65 mM solutions of EDA in: (a) Cyclohexane; (b) THF; (c) ACN; (d) EtOH. The line colours indicate spectra obtained at different time delays shown by the inset key in panel (a). Black arrows show the directions of change in intensity of spectral features over time. The assigned structures of the C-H insertion products are shown in panels (a) – (d). The structures for C-H insertion into THF and EtOH represent one possible product only, with insertion into other C-H bonds also expected.

TRIR spectra in Figure S1(a) for EDA in cyclohexane show the formation of a band at 1745 cm^{-1} which grows with a 12.6 ± 0.4 ps time constant. The absorbing molecules are born with excess internal energy, as is evident from the greater breadth of the bands at early time delays, and the bands shift to higher wavenumber as this excess energy is transferred to the solvent bath. Similar signatures are found in THF at 1739 cm^{-1}, ethanol at 1735 cm^{-1} and a very weak feature in ACN around 1740 cm^{-1}, each of which overlaps a weak negative-going feature located at ~1750 cm^{-1}.

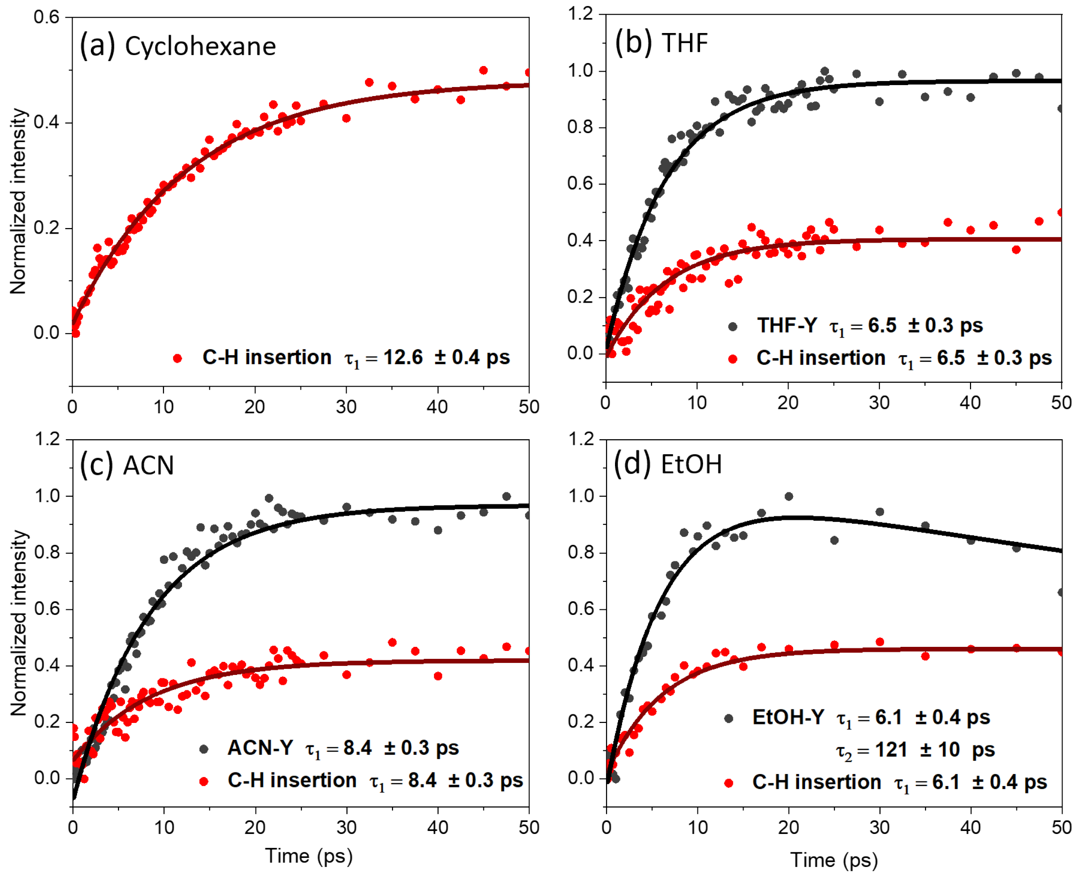

Figure S2 - Time-dependent integrated band intensities (filled circles) and kinetic fits (solid lines) for C-H insertion products (red) and solvent derived ylides (black) following the 270-nm photoexcitation of 65 mM EDA in: (a) Cyclohexane; (b) THF; (c) ACN; (d) EtOH. Within a single panel, all data are globally fitted to single or biexponential functions to obtain the reported time constants. The band intensities for ylides have been normalized to maximum values of 1.0 whereas C-H insertion product band intensities have been normalized to maximum values of 0.5 for clarity.

The assignment of the negative feature at ~1750 cm^{-1} in all the solvents studied is to an EDA ground state bleach (from UV photolytic depletion of the EDA), which is confirmed by observation of a weak band around 1750 cm^{-1} in the steady-state FTIR spectra of EDA in each of the various solvents (see Figure S5). The absorbing species responsible for the growing bands in the TRIR spectra develop in competition with ylide intermediates. Time constants for the changes in band intensity are reported in Table 1. We assign these bands to the C-H insertion products shown in Figure 1(a)–(d) that arise from the reaction of the singlet carbene with solvent molecules. The

magnitudes of the time constants for the growth of these absorption bands in all three solvents suggest rapid reaction of the singlet carbene with a molecule in its first solvent shell. The assignments are supported by previous identification of C-H insertion products after the photoexcitation of EDA in propanol,[1] and the good agreement of the band positions with known IR spectra of possible C-H insertion products (see Table S3).

S1.2 Enol formation

We recently reported the preferential formation of enol intermediates over ylides or carbocations during the reaction of an ethyl diazoacetoacetate derived α-dicarbonyl carbene with alcohols.[2] Here, we present evidence of a similar reaction pathway for the EDA-derived singlet α-carbonyl carbene. Figure S3 shows TRIR and kinetic data for the 270-nm photoexcitation of 65 mM EDA in methanol, in which a transient IR absorption band at 1718 cm^{-1} initially rises with the same time constant as the MeOH-Y band at 1618 cm^{-1}, suggesting a competing reaction pathway of the singlet carbene. The 1718 cm^{-1} band continues to grow with a time constant that matches the decay of the 1618 cm^{-1} feature and the growth of the product ether band at 1745 cm^{-1}. In a prior study, only trace amounts of C-H insertion products were found following EDA photolysis in methanol,[3] with such products experimentally found to show an absorption band at 1735 cm^{-1}.[4] Because of their low yields, such species are not expected to make significant contributions to the intensities of observed bands in the probed wavenumber interval. Moreover, we do not observe C-H insertion products sequentially from ylide-mediated pathways in THF and ACN solutions, hence we discount assignment of the 1718 cm^{-1} band to the C-H insertion product. Instead, we assign this 1718 cm^{-1} band to a mixture of E and Z isomers of an enol, as shown in Scheme S1, with support from the computed carbonyl stretching frequencies of 1714 cm^{-1} (E) and 1720 cm^{-1} (Z). We suggest prompt formation of the E-Enol by the concerted addition of MeOH to carbene (2) by the mechanism proposed in Scheme S2, but discount prompt formation of the Z-Enol on stereochemical grounds. Instead, we propose that the Z-Enol forms by proton transfer within the MeOH-ylide in competition with ether formation. The E-Enol is unlikely to form sequentially from the ylide because delocalization of the negative charge in the MeOH-ylide prevents bond rotation. A corresponding Enol band at 1722 cm^{-1} is similarly observed for EDA photoexcitation in ethanol, and grows with bi-exponential time constants that match the

kinetics observed for the EtOH-Y. Although we do not see evidence for the tautomerization pathways of E and Z Enol isomers, these likely occur over time durations outside our experimental limits. On the basis of the above observations, the proposed mechanism of reaction of the singlet carbene with methanol is summarized in Scheme S1.

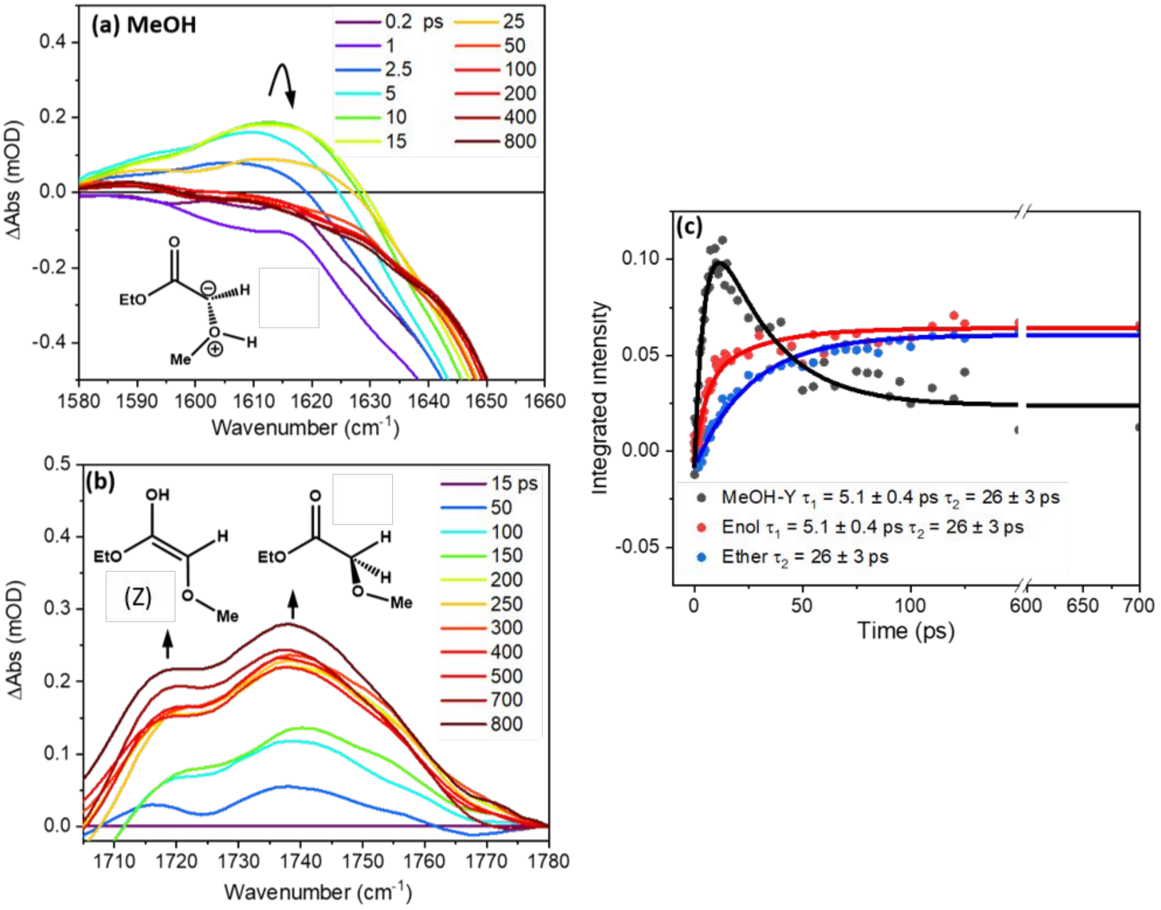

Figure S3 - TRIR spectra for the 270-nm photoexcitation of 65 mM EDA in methanol, in the wavenumber ranges (a) 1580-1660 cm^{-1} and (b) 1705-1780 cm^{-1}. The TRIR spectrum obtained at a time delay of 15 ps has been subtracted from all spectra to remove any possible signatures of C-H insertion products and to highlight the formation of Enol and Ether products at later times. The line colours indicate spectra obtained at different time delays shown by the inset keys. Black arrows show the formation of photoproducts. (c) Time-dependence of integrated band intensities and kinetic fitting for the ylide MeOH-Y (black), Enol (red), and Ether (blue) with the structures shown in panels (a) and (b), and in Scheme S1.

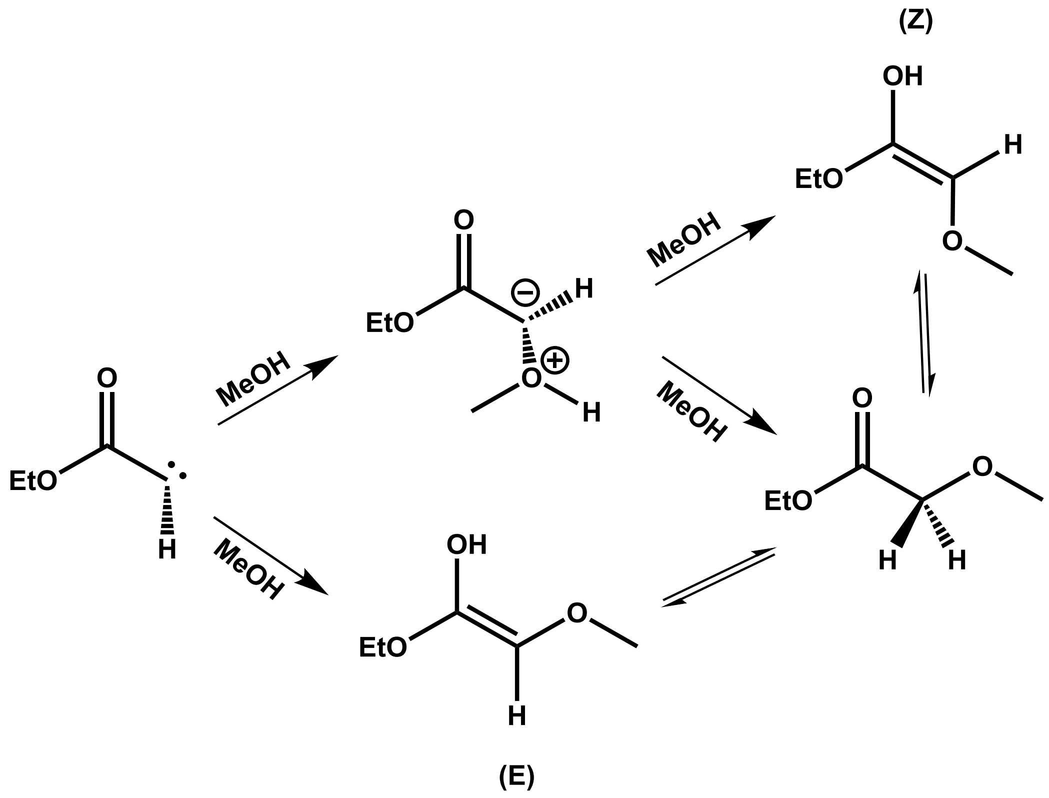

Scheme S1 – Production of E and Z Enol isomers, MeOH-Y, and an Ether from the reaction of the singlet carbene with methanol.

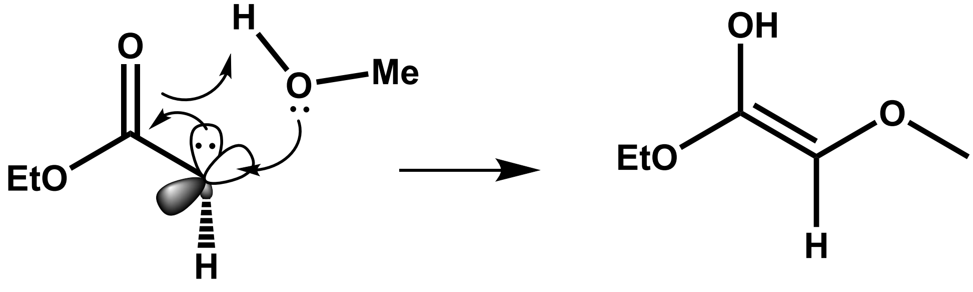

(2)

(E)

Scheme S2 – Proposed mechanism for the concerted addition of MeOH to carbene (2) to form the E-isomer of the Enol.

Section S2 – Supporting FTIR, UV-Vis, and Time resolved infrared spectra

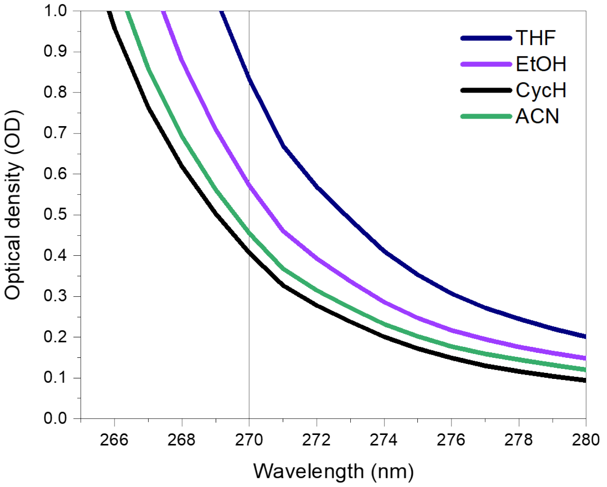

Figure S4 – UV-Vis spectra measured in the 255-280 nm range for 65mM EDA solutions in a cell with a 150 µm pathlength. The solvents used are cyclohexane (CycH, black), ethanol (EtOH, purple), acetonitrile (ACN, green), and tetrahydrofuran (THF, blue). The vertical line shows the photoexcitation wavelength (270 nm) used in TRIR experiments.

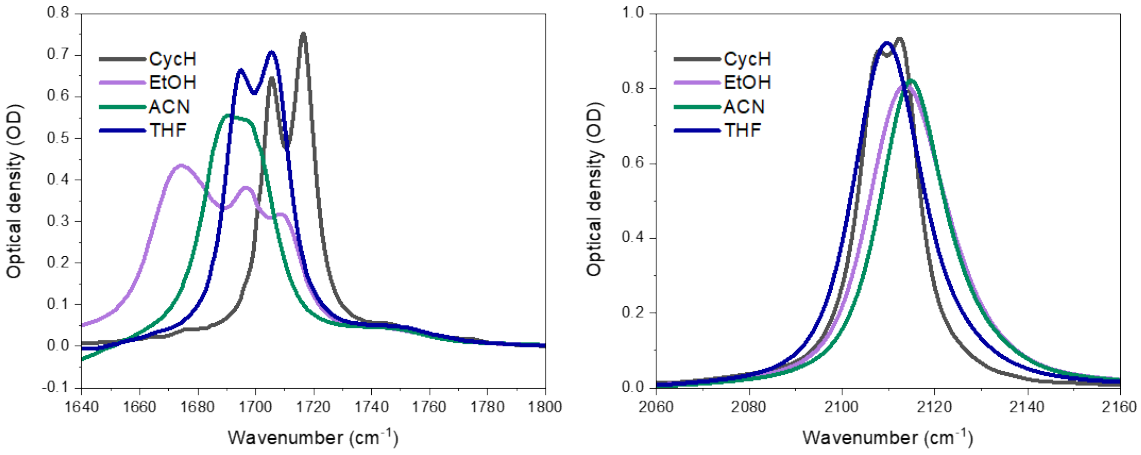

Figure S5 – FTIR spectra measured in the wavenumber range 1640-1800 cm^{-1} (Left) and 2060-2160 cm^{-1} (Right) for 65 mM EDA solutions, using a cell with a 150 µm pathlength. The solvents are cyclohexane (black), ethanol (purple), acetonitrile (green), and tetrahydrofuran (blue).

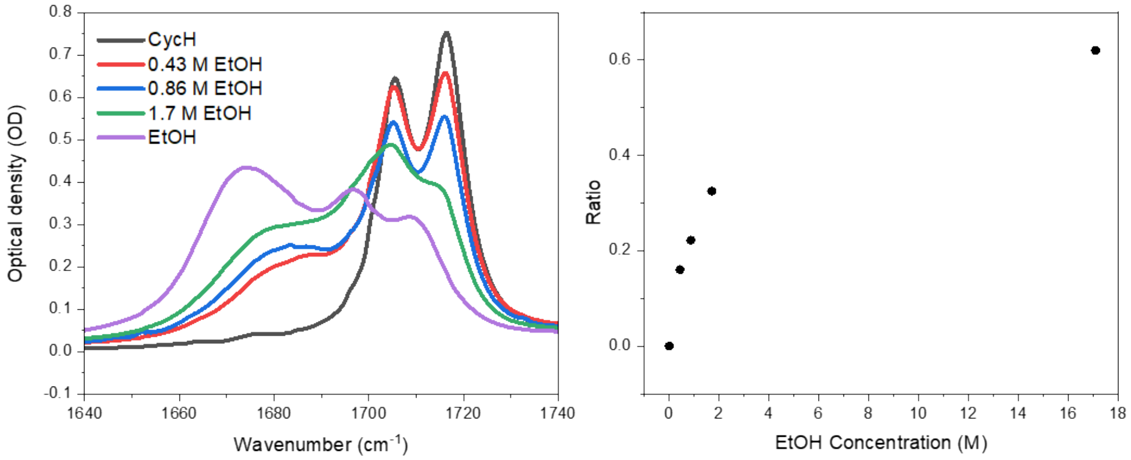

Figure S6 – Left - FTIR spectra measured in the wavenumber range 1640-1740 cm^{-1} for 65 mM EDA solutions in a cell with a 150 µm pathlength and various concentrations of EtOH in CycH. Right – Hydrogen-bonded EDA : uncomplexed EDA band intensity ratio dependence on EtOH concentration. The intensities were taken at the peak of the uncomplexed anti-EDA band, which has its centre between 1710 and 1716 cm^{-1}, and the complexed-EDA band with a centre between 1670 and 1680 cm^{-1}. The inset key shows the solvent compositions.

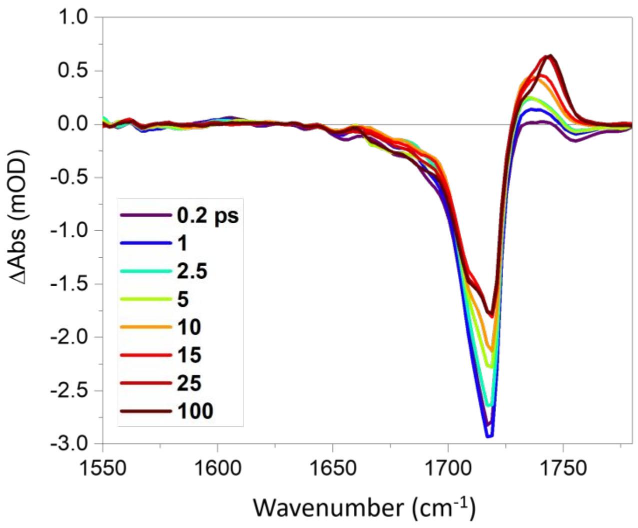

Figure S7 - TRIR spectra obtained at wavenumbers from 1550-1780 cm^{-1} for the photoexcitation of a 65 mM solution of EDA in cyclohexane. Spectra obtained at various time delays are plotted in different colours, as shown by the key. The spectra show no evidence of ylide formation.

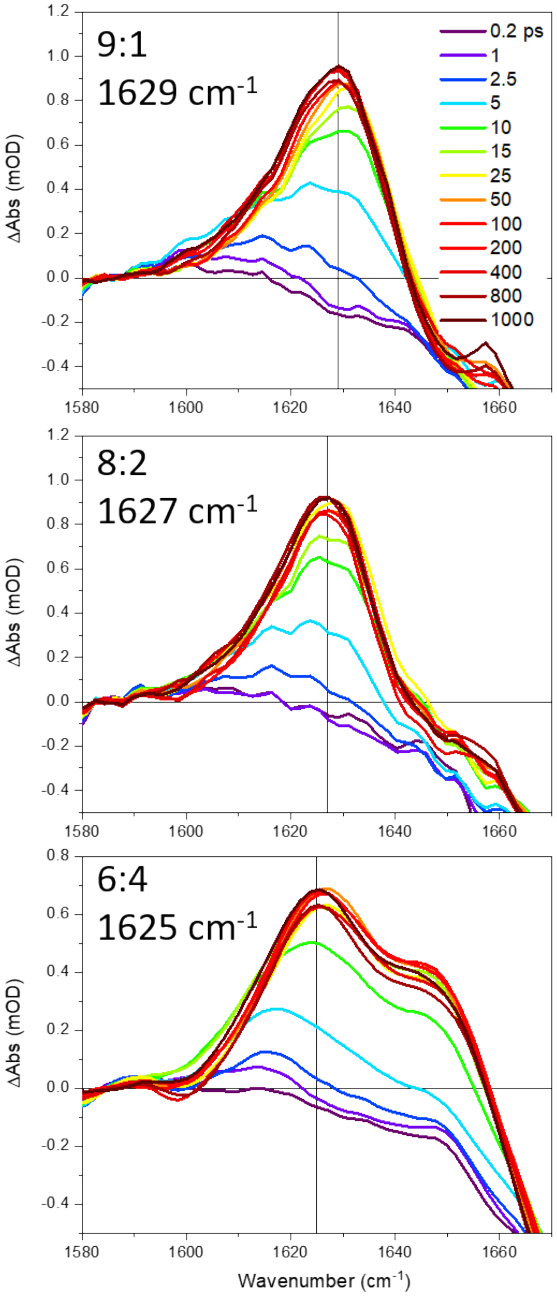

Figure S8 – TRIR spectra obtained in the range 1580-1670 cm^{-1} for the photoexcitation of a 65 mM solution of EDA in mixed solvents of THF:ACN with the specified ratios: Top - 9:1; Middle - 8:2; bottom - 6:4. Spectra obtained at various time delays are plotted in different colours, as shown by the key in panel (a). Vertical lines shown the peak position of the THF-Y band, with the value shown as an inset wavenumber.

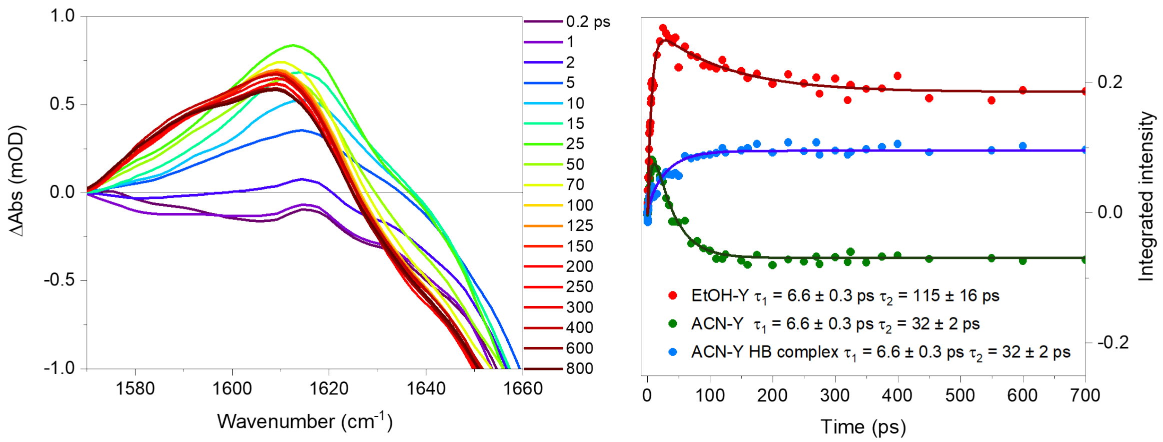

Figure S9 – Left - TRIR spectra obtained at wavenumbers from 1570-1660 cm^{-1} for the photoexcitation of a 65 mM solution of EDA in 9.6M EtOH in ACN. Spectra obtained at various time delays are plotted in different colours, as shown by the key. Right – Kinetic fitting of integrated band intensities of the EtOH-Y (Red, ~1620 cm^{-1}), ACN-Y (Green, ~1640 cm^{-1}) and the ACN-Y HB complex (Blue, ~1610 cm^{-1}). Transient features are more heavily overlapped than for spectra in THF and therefore are more difficult to analyse to extract accurate time constants. Uncertainties are reported from the kinetic fits and do not take into account errors from the spectral decomposition. A similar decomposition was performed as in Figure S11.

Section S3 – TRIR Spectral Decomposition

Spectral decompositions were carried out using the KOALA program,[5] with examples provided here. The spectral decompositions of Ylide (Figure 1) and C-H insertion products (Figures S1 and S3) in neat solvents are illustrated for THF in Figure S10. To extract time-constants for the growth of photoproduct bands, Gaussian functions with variable widths and centres were used to incorporate the effects of vibrational cooling on the time-evolution of photoproduct bands at earlier time delays (0-15 ps). The ground state bleaches (GSB) from photolytic depletion of the EDA precursor appear as negative-going bands in the TRIR spectra, and may be overlapped by the absorption bands of reaction intermediates or products at time delays > 0 ps. To account for these GSB features, our decomposition included an experimentally derived spectral basis function for the shape of the GSB feature; this basis function was extracted from our TRIR spectra obtained at short time delays, before any overlapping ylide or C-H insertion product bands developed. In the spectral fitting, the shape of this band was retained, but its amplitude was allowed to decrease to account for the partial recovery of ground-state EDA. The same procedure was used for analysis of TRIR data obtained for EDA solutions in all the studied solvents.

We observe no changes to the GSB intensities for time delays >15 ps, as is illustrated in Figure S7 for EDA solutions in cyclohexane. We therefore do not expect vibrational cooling and GSB recovery to contribute to the intensities of partially overlapping bands assigned to solvated ylide products, which evolve on longer timescales (e.g., Figures 4 and 6). Hence, the solvated ylide bands discussed in the main paper were fitted to Gaussian functions with fixed widths and centres (see Figures S11 and S12).

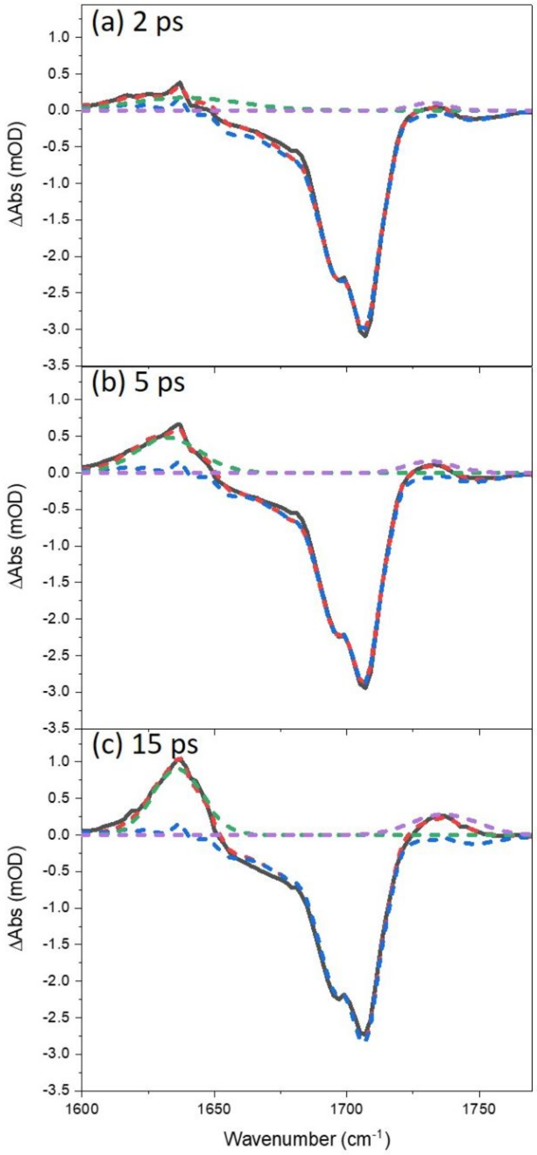

Figure S10 – Example fitting procedure for the decomposition of overlapping bands correpsonding to THF-Y, C-H insertion products, and and the ground state bleach (GSB), observed following the 270-nm photoexcitation of 65 mM EDA in THF. Transient spectra are shown at time delays of: (a) 2 ps; (b) 5 ps; and (c) 15 ps. An early time basis function (Blue) is used to fit the partial recovery of the EDA GSB feature. It is overlapped by Gaussian functions with variable widths and centre wavenumbers to produce the simulated curve (Red) which best describes the experimental data (Black). The Gaussian functions shown account for the THF-Y (Green) and the C-H insertion product (Purple).

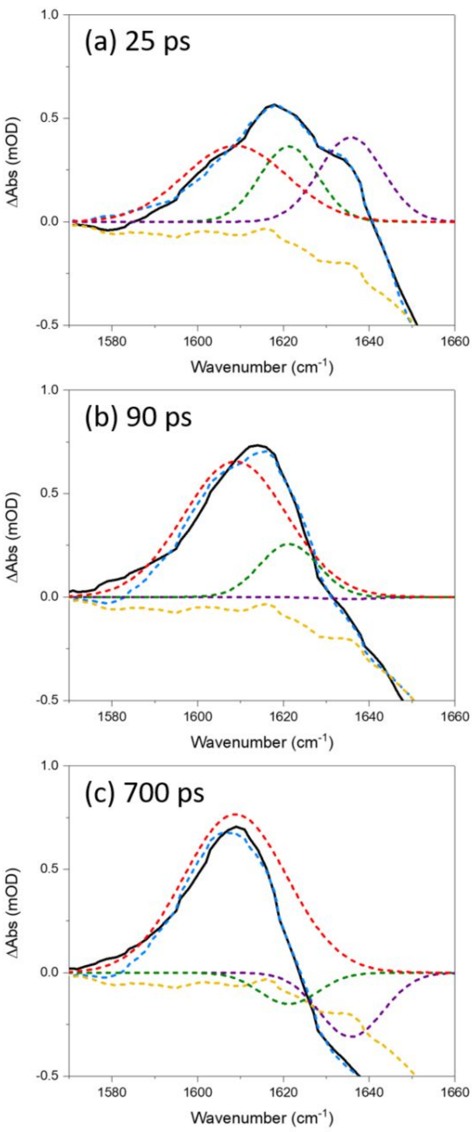

Figure S11 – Example fitting procedure for the decomposition of overlapping EtOH-Y, THF-Y, and THF-Y HB complex bands for the 270-nm photoexcitation of 65 mM EDA with 1.7 M EtOH in THF. Transient spectra are shown at time delays of: (a) 25 ps; (b) 90 ps; and (c) 700 ps. An earlier time basis function (Yellow) fits the GSB recovery of EDA, and is overlapped with Gaussian functions with fixed widths and centres to produce the simulated curve (Blue) which best describes the experimental data (Black). Gaussian functions are shown for THF-Y (Purple, 1636 cm^{-1}), EtOH-Y (Green, 1619 cm^{-1}), and the THF-Y complex (Red, 1609 cm^{-1}). Some of these fitting functions appear negative at later time delays, possibly because the basis function used to fit the GSB contains a spectral contribution of the ylides formed promptly from reaction with the excited state of EDA.

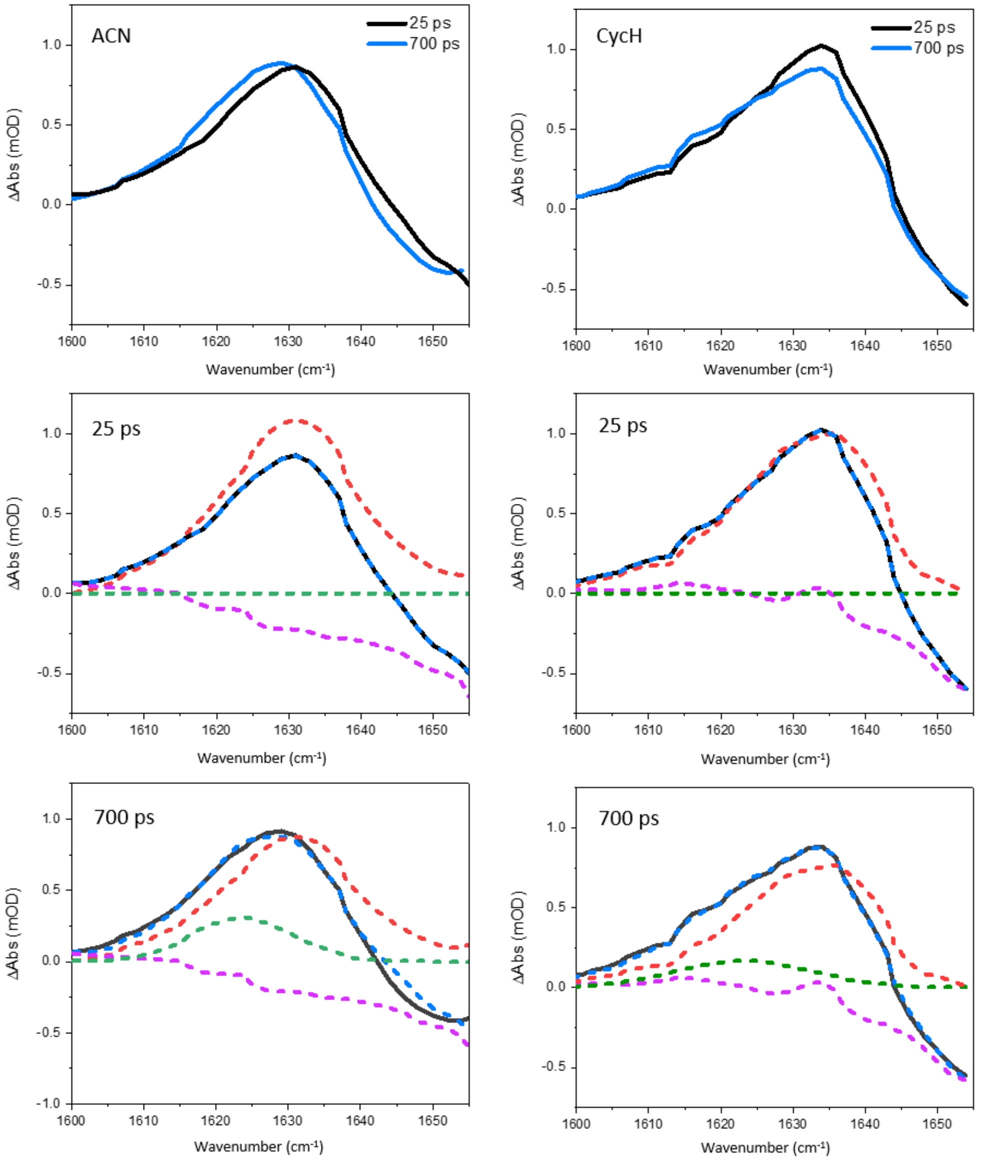

Figure S12 – Example fitting procedure for the decomposition of overlapping THF-Y and THF-Y HB complex bands for the 270-nm photoexcitation of 65 mM EDA with 1.9 M ACN in THF (Left) and 0.46 M Cyclohexane in THF (Right). Transient spectra taken at 25 ps (Solid black line) and 700 ps (Solid blue line) are shown in the top panels and their spectral decomposition in the middle and bottom panels respectively. THF-Y basis functions (Dashed red line) were determined by subtraction of spectra at 0.3 ps (Dashed purple line, a period before THF-Y forms) from spectra taken at 25 ps (after THF-Y forms but before complexation with HB donors) and their amplitudes were allowed to evolve with time. The THF-Y HB complex bands are fitted to Gaussian basis functions (Dashed green lines) with fixed widths and centres to produce the simulated curve (Dashed blue line) which best describes the experimental data.

Section S4 – Supporting Kinetic Fits

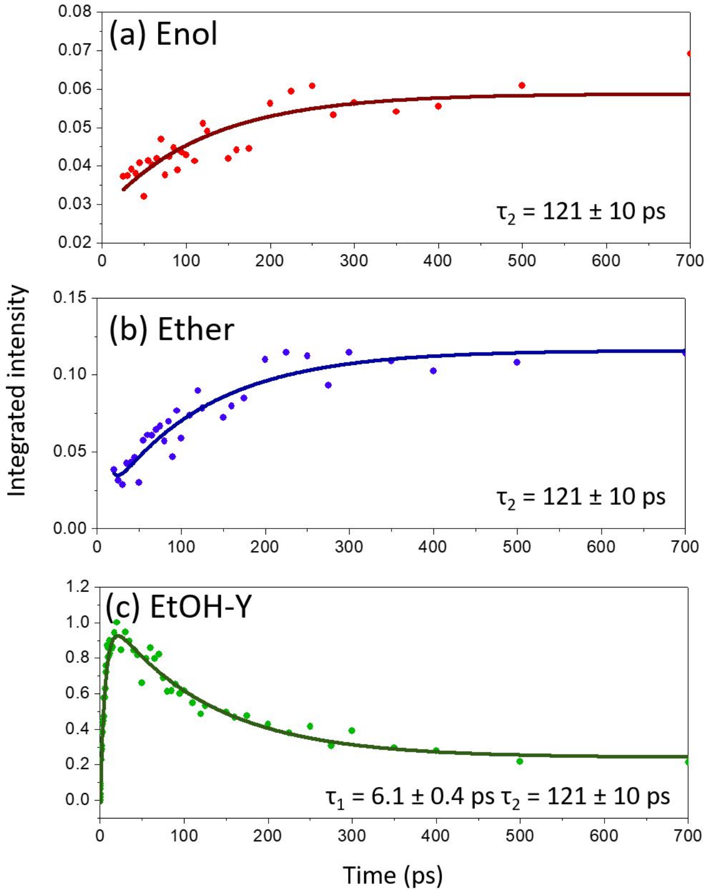

Figure S13 – Time-dependence of integrated band intensities (filled circles) and kinetic fits (solid lines) for a 17.2 M (neat) solution of EtOH. (a) Enol (b) Ether (c) EtOH-Y. The growth of Ether and Enol bands and the decay of the EtOH-Y band intensities have been fitted to the same time constant.

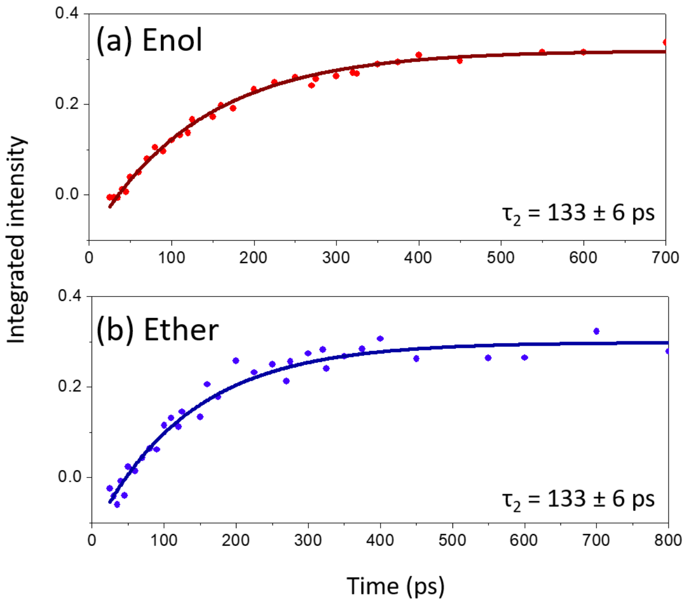

Figure S14 – Time-dependence of integrated band intensities (filled circles) and kinetic fits (solid lines) for a 16.7 M solution of EtOH in THF. (a) Enol (b) Ether. Ether and Enol bands have been fitted to the same time constant.

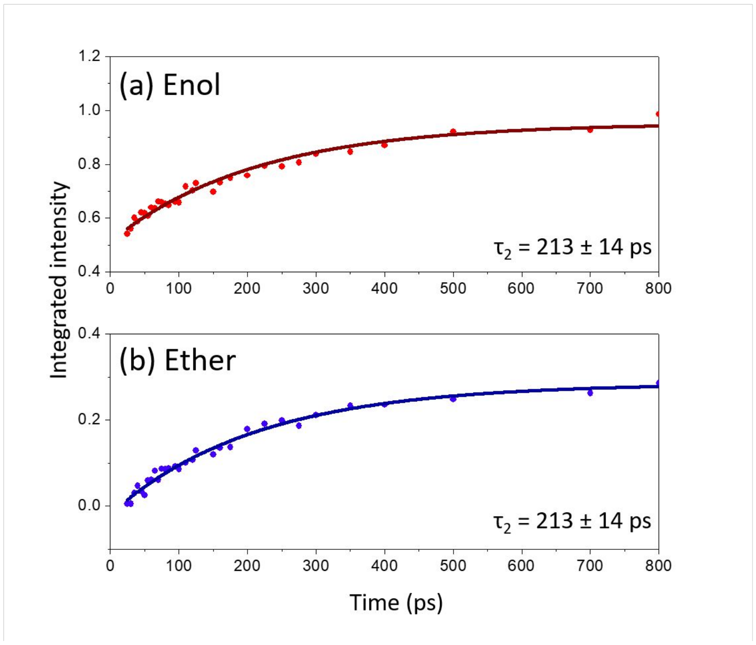

Figure S15 – Time-dependence of integrated band intensities (filled circles) and kinetic fits (solid lines) for a 13.7 M solution of EtOH in THF. (a) Enol (b) Ether. Ether and Enol bands have been fitted to the same time constant.

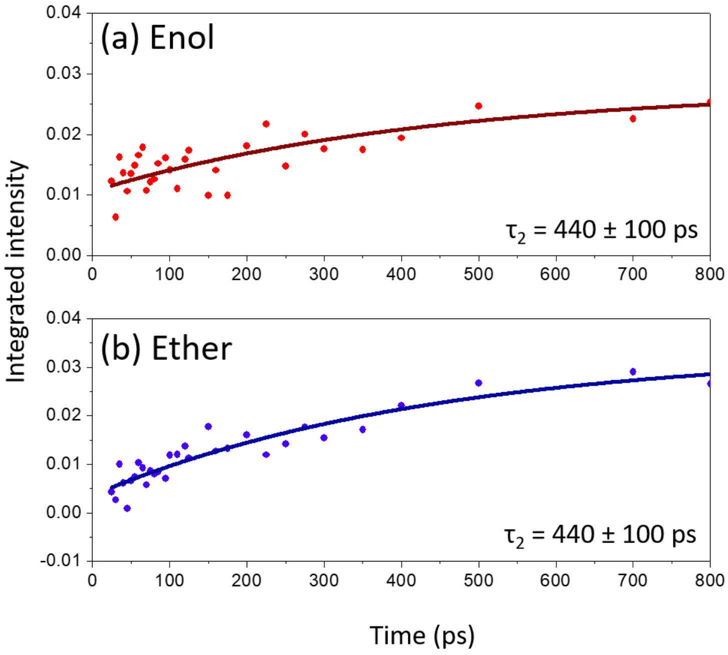

Figure S16 – Time-dependence of integrated band intensities (filled circles) and kinetic fits (solid lines) for an 8.6 M solution of EtOH in THF. (a) Enol (b) Ether. Ether and Enol bands have been fitted to the same time constant.

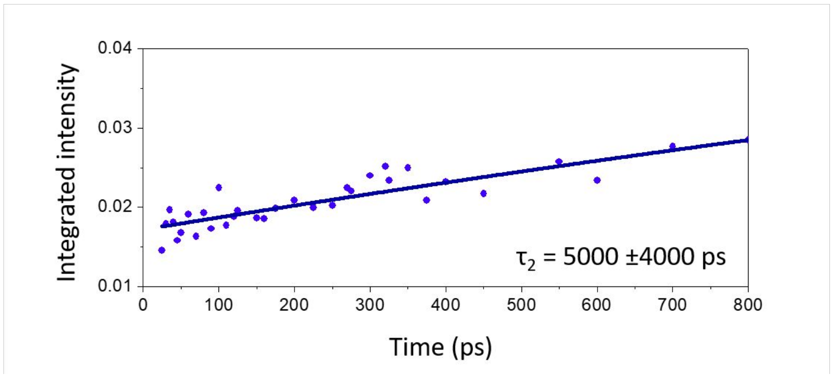

Figure S17 – Time-dependence of the Ether integrated band intensity (filled circles) and the kinetic fit (solid lines) for a 1.7 M solution of EtOH in THF. Enol bands were not detected at this low concentration of EtOH. A time constant greater than 1 ns was obtained, and is estimated to be 5000 ± 4000 ps.

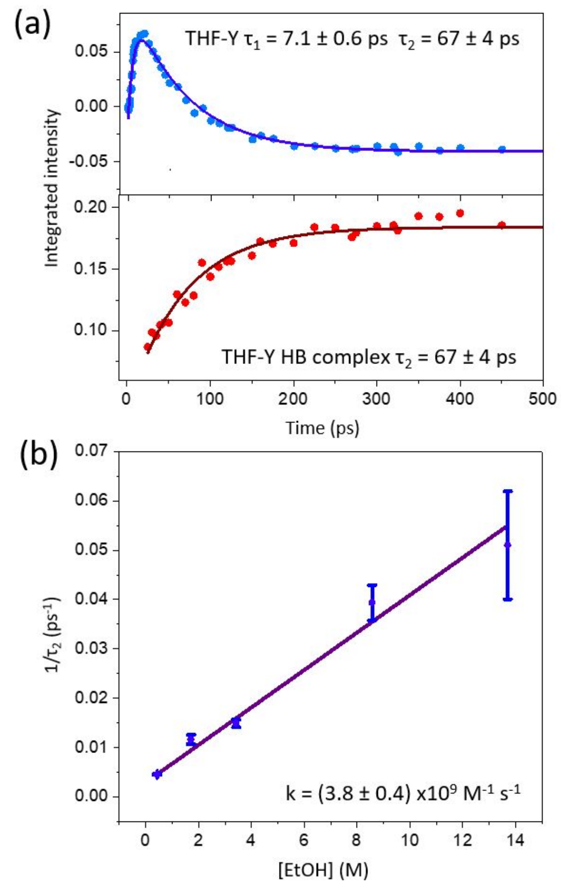

Figure S18 – Kinetics of photoproduct formation after 270-nm photoexcitation of 65 mM EDA in a mixed solution of 3.4 M EtOH in THF. (a) Integrated intensities of TRIR bands (filled circles) assigned to the THF-Y (blue) and THF-Y HB complex (red), and biexponential fits (solid lines). For the THF-Y HB complex, only data points for time delays greater than 25 ps are plotted because at earlier times the TRIR bands overlap those from vibrationally hot ylides. (b) Dependence on the concentration of EtOH of the pseudo-first order rate coefficients ($1/\tau_2$) for the growth of the THF-Y HB complex. Error bars (blue) on experimental data points show the uncertainties from the kinetic fits but do not take into account errors that arise from the spectral decomposition. The gradient of the linear fit (purple) gives the bimolecular rate coefficient. Larger uncertainties at higher concentrations of EtOH result from the low yield of THF-Y because of competitive formation of EtOH-Y.

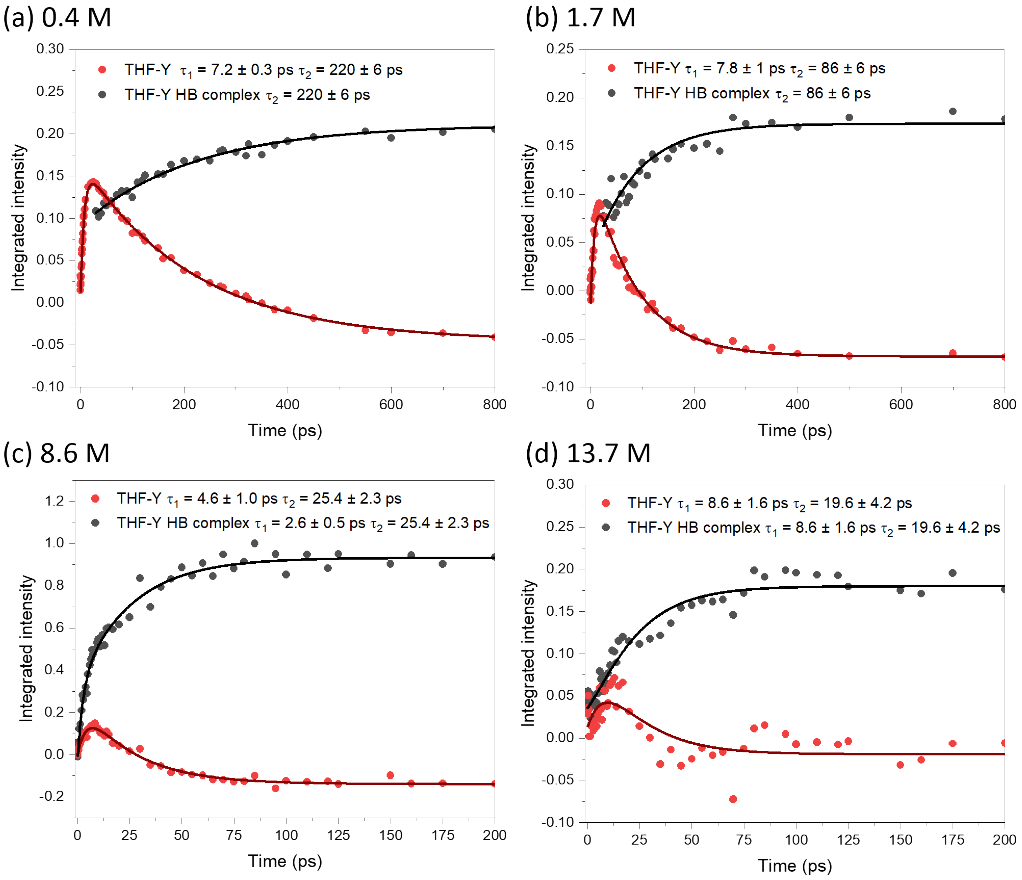

Figure S19 - Time-dependence of integrated band intensities (filled circles) and kinetic fits (solid lines) for THF-Y and the THF-Y HB complex for EtOH:THF solvent mixtures. The concentration of EtOH is reported in each panel. Data for the THF-Y HB complex are plotted for time delays greater than 25 ps in panels (a) and (b) because of overlapping absorption from vibrationally excited THF-Y molecules undergoing vibrational relaxation. Time delays less than 25 ps are included in panels (c) and (d) and the kinetic fits because the time constant for complexation is of similar magnitude to those for the initial formation of THF-Y and vibrational cooling. The greater time constant for the growth of the THF-Y HB band is likely to include components from THF-Y vibrational cooling dynamics and complexation of the vibrationally hot THF-Y molecules with EtOH. Only the larger time constant has been used to determine the bimolecular rate coefficient included in Figure S18. Panels (c) and (d) have been plotted only to time delays of 200 ps because the bands overlap with the slower decay of the EtOH-Y band. We have not performed a kinetic analysis for EtOH-Y because it overlaps and is centred between the THF-Y and THF-Y HB bands, but the EtOH-Y transformation can be monitored using the enol and ether bands which are reported in Figures S13-S17 and discussed in section S1.2 and section 3.2 of the main manuscript.

Section S5 – Computational

The majority of vibrational frequencies were computed at the MP2/6-311++G(d,p) level of theory with inclusion of the stated solvent using the PCM method. Harmonic frequencies were scaled by a factor of 0.9827 to account for anharmonicity. The anharmonicity scaling factor was computed for the syn isomer of EDA and the average applied to all other species. For the singlet and triplet carbenes the CCSD/6-311++G(d,p) level of theory was used with inclusion of the stated solvent using the PCM method. The harmonic frequencies of the computed singlet and triplet carbene bands were corrected for anharmonicity by computing Syn-EDA frequencies at the CCSD/6-311++G(d,p) level of theory and comparing to experimentally determined frequencies to determine the anharmonic scaling factor of 0.9619.

Table S1 – Computed and Observed Vibrational Wavenumbers of EDA Isomers in Various Solvents.

Species	Solvent	Computed vibrational wavenumber / cm^{-1}	Observed vibrational wavenumber / cm^{-1}
Syn-EDA	Cyclohexane	1710	1705
Anti-EDA	Cyclohexane	1725	1716
Syn-EDA	THF	-	1695
Anti-EDA	THF	-	1705
Syn-EDA	ACN	-	1688
Anti-EDA	ACN	-	1700
Syn-EDA	EtOH	-	1696
Anti-EDA	EtOH	-	1709
HB-EDA	EtOH	-	1674

Accurate computation of the properties of singlet carbenes often requires multi-reference methods because of their low lying singlet and triplet excited states. Single-reference calculations were shown to be inadequate for accurate geometries and vibrational frequencies for non-substituted carbenes.[6] However, the bonding in α-carbonyl carbenes reduces their multi-reference character, and these species can therefore be described satisfactorily by single-reference methods. For example, the CCSD(T) level of theory gives reasonable results for non-conjugated substituted carbenes.[7-8] Here, we predict α-carbonyl carbene band frequencies at the CCSD/6-311++G(d,p) level of theory which should be reasonable for the conjugated α-carbonyl carbene.

Table S2 – Computed Vibrational Wavenumbers of Carbene Intermediates.

Species	Solvent	Computed vibrational wavenumber / cm^{-1}	Observed vibrational wavenumber / cm^{-1}
Singlet Carbene	Cyclohexane	1654[a]	-
Triplet Carbene	Cyclohexane	1664[a]	-

[a]Computed at the CCSD/6-311++G(d,p) level of theory with inclusion of a PCM treatment of solvent and scaled by a factor of 0.9619 (obtained as described in the text).

Table S3 – Reported Vibrational Wavenumbers from Prior End-Product Analysis and Observed Vibrational Wavenumbers from the TRIR Measurements in the Current Work for C-H Insertion Products Formed in Various Solvents.

Species	Solvent	Vibrational wavenumber / cm^{-1} (prior product analysis)	Vibrational wavenumber / cm^{-1} (TRIR)
C-H insertion	Cyclohexane	1740[9]	1745
C-H insertion	THF	1738[10]	1739
C-H insertion	EtOH	1735[4]	1735
C-H insertion	MeOH	1735[4]	-
C-H insertion	ACN	-	1740

Note: The small discrepancies between the previously reported and currently observed vibrational wavenumbers of C-H insertion products are due to different bulk solvent environments used.

Table S4 – Computed and Observed Vibrational Wavenumbers of Ylide and Enol Intermediates Formed in Various Solvents.

Species	Solvent	Computed vibrational wavenumber / cm^{-1}	Observed vibrational wavenumber / cm^{-1}
Anti-THF-Y	THF	1642	1636
Syn-THF-Y	THF	1634	1636
Anti-ACN-Y	ACN	1643	1642
Syn-ACN-Y	ACN	1638	1642
Anti-EtOH-Y	EtOH	-	1617
Anti-MeOH-Y	MeOH	1628	1618
E-Enol	MeOH	1714[a]	1718
Z-Enol	MeOH	1720[a]	1718
Enols	EtOH	-	1722
Z-Enolate	MeOH	1618	-
E-Enolate	MeOH	1644	-

[a] Calculated using an anharmonicity scaling factor of 0.9754. The anharmonicity scaling factor was computed for the E-Enol in the gas phase, and was applied to the Enol isomers computed with a PCM treatment of solvent interactions. A different anharmonic correction is used for the Enol because the normal mode assigned within the probing region (1580-1780 cm^{-1}) is a C=C stretching mode, not the conjugated carbonyl groups of ylide and enolate photoproducts.

Table S5 – Computed and Observed Vibrational Wavenumbers of Hydrogen-Bonded Complexes of THF-Y with Various Hydrogen-Bond Donors

Hydrogen-bond donor	Solvent	Computed vibrational wavenumber / cm^{-1}	Observed vibrational wavenumber / cm^{-1}
EtOH	THF	1623	1617
EtOH	EtOH	1614	1608
Chloroform	THF	-	1622
DCM	THF	-	1622
ACN	THF	-	1624
Cyclohexane	THF	-	1625

Full computational details and data files are available at the University of Bristol data repository, data.bris, at https://doi.org/10.5523/bris.38zbrp39vx4az2dmvyzm1440ud.

References

1. Strausz, O. P.; Thap, D. M.; Gunning, H. E. Rearrangement and Polar Reaction of Carbethoxymethylene in 2-Propanol. *J. Am. Chem. Soc.* **1968,** *90*, 1660-1661.
2. Phelps, R.; Orr-Ewing, A. J. Direct Observation of Ylide and Enol Intermediates Formed in Competition with Wolff Rearrangement of Photoexcited Ethyl Diazoacetoacetate. *J. Am. Chem. Soc.* **2020,** *142*, 7836-7844.
3. Chaimovich, H.; Vaughan, R. J.; Westheimer, F. H. Rearrangement accompanying the photolysis of diazoacyl esters. *J. Am. Chem. Soc.* **1968,** *90*, 4088-4093.
4. Drioli, S.; Nitti, P.; Pitacco, G.; Tossut, L.; Valentin, E. Enantiomerically pure tetrahydro-5-oxo-2-furancarboxylic esters from dialkyl 2-oxoglutarates. *Tetrahedron: Asymmetry* **1999,** *10*, 2713-2728.
5. Grubb, M. P.; Orr-Ewing, A. J.; Ashfold, M. N. R. KOALA: A program for the processing and decomposition of transient spectra. *Rev. Sci. Instrum.* **2014,** *85*, 064104.
6. Hajgató, B.; Nguyen, H. M. T.; Veszprémi, T.; Nguyen, M. T. Triplet–singlet energy gaps in iodo-carbenes (I–C–X): Remarkable discrepancy between theory and experiment. *Phys. Chem. Chem. Phys.* **2000,** *2*, 5041-5045.
7. Matzinger, S.; Fuelscher, M. P. Methyl Substitution in Carbenes. A Theoretical Prediction of the Singlet-Triplet Energy Separation of Dimethylcarbene. *J. Phys. Chem.* **1995,** *99*, 10747-10751.
8. Wang, X.; Agarwal, J.; Schaefer III, H. F. Characterizing a nonclassical carbene with coupled cluster methods: cyclobutylidene. *Phys. Chem. Chem. Phys.* **2016,** *18*, 24560-24568.
9. Upadhya, T. T.; Sudalai, A. A short and efficient enantioselective synthesis of cyclohexylnorstatine, a key component of a renin inhibitor. *Tetrahedron: Asymmetry* **1997,** *8*, 3685-3689.
10. Bellur, E.; Freifeld, I.; Böttcher, D.; Bornscheuer, U. T.; Langer, P. Synthesis of (tetrahydrofuran-2-yl)acetates based on a 'cyclization/hydrogenation/enzymatic kinetic resolution' strategy. *Tetrahedron* **2006,** *62*, 7132-7139.
